# Exploring the diversity, bioactivity of endophytes, and metabolome in *Synsepalum dulcificum*

**DOI:** 10.3389/fmicb.2024.1258208

**Published:** 2024-02-27

**Authors:** Sisi Liu, Yage Hou, Kaixuan Zheng, Qian Ma, Meng Wen, Shicheng Shao, Shaohua Wu

**Affiliations:** ^1^Key Laboratory for Southwest Microbial Diversity of the Ministry of Education, Yunnan Institute of Microbiology, School of Life Sciences, Yunnan University, Kunming, China; ^2^Department of Gardening and Horticulture, Xishuangbanna Tropical Botanical Garden, Chinese Academy of Sciences, Mengla County, Yunnan, China

**Keywords:** *Synsepalum dulcificum*, microbiome, metabolomics, endophytes, diversity, biological activities

## Abstract

*Synsepalum dulcificum* exhibits high edible and medicinal value; however, there have been no reports on the exploration of its endophyte resources. Here, we conducted analyses encompassing plant metabolomics, microbial diversity, and the biological activities of endophytic metabolites in *S*. *dulcificum*. High-throughput sequencing identified 4,913 endophytic fungal amplicon sequence variants (ASVs) and 1,703 endophytic bacterial ASVs from the roots, stems, leaves, flowers, and fruits of *S. dulcificum*. Fungi were classified into 5 phyla, 24 classes, 75 orders, 170 families, and 313 genera, while bacteria belonged to 21 phyla, 47 classes, 93 orders, 145 families, and 232 genera. Furthermore, there were significant differences in the composition and content of metabolites in different tissues of *S*. *dulcificum*. Spearman’s correlation analysis of the differential metabolites and endophytes revealed that the community composition of the endophytes correlated with plant-rich metabolites. The internal transcribed spacer sequences of 105 isolates were determined, and phylogenetic analyses revealed that these fungi were distributed into three phyla (Ascomycota, Basidiomycota, and Mucoromycota) and 20 genera. Moreover, 16S rDNA sequencing of 46 bacteria revealed they were distributed in 16 genera in three phyla: *Actinobacteria, Proteobacteria, and Firmicutes*. The antimicrobial activities (filter paper method) and antioxidant activity (DPPH and ABTS assays) of crude extracts obtained from 68 fungal and 20 bacterial strains cultured in different media were evaluated. Additionally, the α-glucosidase inhibitory activity of the fungal extracts was examined. The results showed that 88.6% of the strains exhibited antimicrobial activity, 55.7% exhibited antioxidant activity, and 85% of the fungi exhibited α-glucosidase inhibitory activity. The research suggested that the endophytes of *S*. *dulcificum* are highly diverse and have the potential to produce bioactive metabolites, providing abundant species resources for developing antibiotics, antioxidants and hypoglycemic drugs.

## Introduction

Endophytic communities are a highly diverse polyphyletic group that inhabit plant organs but do not cause considerable damage to the host (Sun et al., 2020; Zhang et al., 2021). Endophytes can impact various aspects of plant responses, such as enhancing nutrient availability and increasing tolerance to both biotic and abiotic stresses through the production of specific specialized metabolites (Bacon, 2000; Nagabhyru et al., 2013). At present, studies have reported that endophytes can be isolated from all plant species and each plant may contain at least one or more endophytes (Zhang et al., 2020). Furthermore, there are various plants, including liverworts, mosses, lycophytes, equisetopsids, ferns, and seed plants (Ezra et al., 2004; Hyde and Soytong, 2008). Endophytic species differ based on different host plants; in addition, the same plant may have different endophytic species owing to different growth environments (Compant et al., 2021). Among them, the predominant endophytic fungi are *Diaporthales* in angiosperms and *Helotiales* in gymnosperms (Sieber, 2007). They are generally found in the aboveground parts of the host plant, including leaves, stems, and flowers (Saikkonen et al., 1998). Most endophytic bacteria belong to the genus *Streptomyces*, *Pseudomonas*, *Bacillus* and *Enterobacter* (Trivedi et al., 2020). Furthermore, among the different tissues of woody plants, endophytic *Actinomycetes* are the most abundant in the roots, followed by stems and leaves. Endophytes are one of the most important components of decomposers, symbionts, and pathogens in the ecosystem, and a balanced symbiotic continuum exists between the endophytes and host (Debbab et al., 2011; Matsuoka et al., 2021). There are diverse and several parasitic endophytes in medicinal plants; their secondary metabolites have rich structural types and obvious biological activities (Shen et al., 2023). Moreover, as a potential source of biologically active natural products, many researchers are focusing on the development and mining of endophytic resources from medicinal plants.

In China, Xishuangbanna is an important area for terrestrial biodiversity conservation as well as a base for the development and use of tropical plant resources (Tang et al., 2006). Because this region is characterized by constantly high temperatures and rainfall as well as strong plant photosynthesis and transpiration, the invasion and colonization of endophytes have become more favorable (Chen et al., 2018). However, there are still relatively few studies on the plant endophytes in this region. *S*. *dulcificum* belongs to the family *Sapotaceae*; after the 1960s, it was introduced to Hainan, Sichuan, Yunnan (Xishuangbanna), and other places in China for cultivation (Tchokponhoue et al., 2021). The extracts of *S*. *dulcificum* exhibit several biological activities, including antioxidant, antimicrobial, antitumor, cholesterol-lowing, and antidiabetic effects (Inglett and Chen, 2011; Yang et al., 2022). However, studies on the endophytes of *S. dulcificum* remain scarce. Plant endophytes, an important source of new natural products, can produce biologically active substances similar to their host plants (Srinivasa et al., 2022). Therefore, exploring the endophytic resources from tropical plants will be of great significance.

At present, advances in science and technology have led to the increased application of combined multiomics analysis to solve complex biological problems (Yang et al., 2023). Among them, the combined analysis of the microbiome and metabolome has had positive implications in studies on medicinal plants (Li et al., 2022). In the present study, we identified the communities and diversity of uncultured and culturable endophytes from different tissues of *S*. *dulcificum*. In addition, by analyzing five tissue microbiomes and untargeted metabolomes of *S*. *dulcificum*, we identified the relationship between different tissue metabolites and their microbial composition. Moreover, we determined the antimicrobial, antioxidant and α-glucosidase inhibitory activities of the crude extracts of 68 fungi and 20 bacteria to identify the endophytic species with different biological activities. Our study will provide a preliminary basis for studies on the endophytes from *S*. *dulcificum* and contribute to the research of antimicrobial agents, natural antioxidants and hypoglycemic drugs.

## Materials and methods

### Plant materials

**FIGURE 1:**
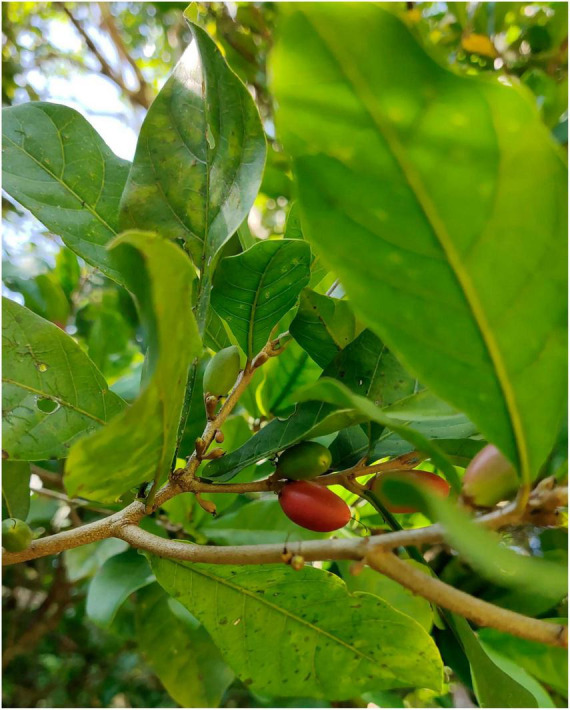
Pictures of selected plants from Xishuangbanna.

The healthy plant roots, stems, leaves, flowers, and fruits of *S*. *dulcificum* were collected from the same plantation in Xishuangbanna Prefecture (21°8′–22°36′ N, 99°56′–101°51′ E), Yunnan, China (Figure 1). The sampled plants were of the same age, exhibited no noticeable signs of disease infestation or abnormal growth, and were capable of flowering and fruiting normally. The plant materials were promptly frozen and transported to Kunming immediately after collection. Samples designated for omics analysis were pre-processed according to the testing company’s specifications, immediately frozen in liquid nitrogen, and then stored at −80°C. Samples for pure culture experiments were immediately subjected to endophytes isolation. The remaining samples were frozen with liquid nitrogen and stored at the Yunnan Institute of Microbiology.

### Untargeted metabolomic analysis

A hundred milligrams of samples from each of the six biological replicates of roots, stems, leaves, flowers, and fruits were extracted with 1 ml of precooled 50% methanol, vortexed for 1 min, and incubated at room temperature for 10 min. The extraction mixture was then stored overnight at −20°C. After centrifugation at 4,000 g for 20 min, the supernatants were transferred into new 96-well plates. A liquid chromatography–mass spectrometry (LC–MS) system was used to collect sample information according to the instructions of the system. All chromatographic separations were performed using the Vanquish Flex UHPLC system (Thermo Fisher Scientific, Bremen, Germany). The metabolites eluted from the column were detected using the Q-Exactive (Thermo Scientific) high-resolution tandem mass spectrometer. LC–MS raw data files were converted into mzXML format and then processed by the XCMS, CAMERA, and MetaX toolboxes implemented with R software (Smith et al., 2006; Kuhl et al., 2012; Wen et al., 2017). The metabolites were annotated using the online Kyoto Encyclopedia of Genes and Genomes (KEGG) and Human Metabolome Database (HMDB) databases. Student’s *t*-tests were conducted to elucidate differences in the metabolite concentrations between the two phenotypes. The p-value was adjusted for multiple tests using the false discovery rate (FDR). Furthermore, partial least squares-discriminant analysis (PLS-DA) was conducted using MetaX to discriminate the different variables between the groups. Then, the VIP value was calculated. A VIP cutoff value of 1.0 was used to select important features (Ke et al., 2021; Li et al., 2021). Metabolome sequencing was conducted by Hangzhou Lianchuan Biotechnology Co., Ltd.

### High-throughput sequencing and statistical analysis

Total DNA was extracted from different tissue samples of *S*. *dulcificum* using the cetyltrimethylammonium bromide (CTAB) method (Logue et al., 2016). DNA quality was assessed using agarose gel electrophoresis, and DNA was quantified using an ultraviolet spectrophotometer. The internal transcribed spacer (ITS) region was amplified using the primers ITS1FI2 (5′-GTGARTCATCGAATCTTTG-3′) and ITS2 (5′-TCCTCCGCTTATTGATATGC-3′), and the 16S region was amplified using the primers F (5′-GTGCCAGCMGCCGCGG-3′) and R (5′-CCGTCAATTCMTTTRAGTTT-3′) (Walters et al., 2015). Polymerase chain reaction (PCR) products were purified using AMPure XT beads (Beckman Coulter Genomics, Danvers, MA, USA) and quantified using Qubit (Invitrogen, USA). Amplicon pools were prepared for sequencing, and the size and quantity of the amplicon library were assessed using the Agilent 2100 Bioanalyzer (Agilent, USA) and Library Quantification Kit for Illumina (Kapa Biosciences, Woburn, MA, USA), respectively. The libraries were sequenced on the NovaSeq PE250 platform.

Chimeric sequences were filtered using Vsearch software (v2.3.4). After dereplication using DADA2, feature tables, and sequences were obtained. Then, alpha and beta diversities were calculated using QIIME2; the diagrams were drawn using the R package (v3.5.2). Basic Local Alignment Search Tool (BLAST) was used for sequence alignment, and the feature sequences were annotated using the SILVA database for each representative sequence. Furthermore, species annotation was performed using Ribosomal Database Project based on the ASV file. Species abundance in each sample was counted using the ASV abundance table. Chao1, Observed species, Good’s coverage, Shannon, Simpson, and Pielou-e indices were calculated to determine the abundance and evenness of the endophytes in *S*. *dulcificum*. All experimental data are presented as the mean ± SD derived from six independent observations. The data were subjected to one-way analysis of variance (ANOVA) followed by Duncan’s multiple range tests using SPSS 19.0 software. P < 0.05 was used to define statistically significant differences between the control and experimental groups. Additionally, PLS-DA (VIP > 1) and Anosim analyses were conducted to observe differences among various tissue samples.

### Joint analyses of metabolome and microbiome

To investigate the correlation between differential endophytes and plant metabolites, we performed Spearman’s correlation analysis on differential endophytes and metabolites among different tissues. Differential endophytes and metabolites with a correlation greater than 0.8 and significant correlation of p < 0.05 were selected. The analysis was carried out using the R programming language, version 3.4.4 (Li et al., 2016).

### Isolation of culturable endophytes

The roots, stems, leaves, flowers, and fruits of *S*. *dulcificum* were surface sterilized using the method described by Zhou et al., with some modifications (Zhou et al., 2018). Endophytic fungi were isolated using the method described by Ezra et al. (2004). Plant tissues were initially washed with tap water, immersed in 75% ethanol for 30–60 s, rinsed two times with sterile distilled water, rinsed with 2% sodium hypochlorite for 1.5–3 min, and finally rinsed with sterile distilled water three times. Different materials had different sterilization times (roots > stems > fruits > leaves > flowers). Sterilization was conducted within a super-clean workbench. After sterilization, these tissues were dried with sterile paper and then cut into small pieces of 1 × 1 cm. One hundred and fifty fragments from each of roots, stems, leaves, flowers and fruits were used for fungal isolation. Four isolation media were used in this experiment: potato dextrose agar (PDA), modified Martin’s agar (MRS), corn meal agar (CMA), and complete yeast medium (CYM). These media were supplemented with chloramphenicol (Supplementary Table 1). During the procedure, plates containing PDA medium were openly placed within the super-clean workbench, serving as control: 1. 0.2 mL of sterile water from the final wash of plant samples was inoculated into PDA medium as control; 2. After incubation at 28°C for 2–4 weeks, no colonies grew on the control plates, indicating successful surface disinfection. These fragments were cultured at 28°C for 2–4 weeks, and the growth of fungal hyphae was observed every day. The hyphal tip method was used to purify the culture, and the purity of the culture was determined based on colony morphology (Kusari et al., 2013).

The sterilized plant tissues were cut into small pieces and placed into centrifuge tubes used to isolate endophytic bacteria. Phosphoric acid buffer (1%) was added into the centrifuge tubes and homogenized for 5 min under sterile conditions (8000 rpm). The homogenized plant samples were shaken at 200 rpm for 1 h and then diluted and coated on the separation medium and cultured at 28°C for 2–8 weeks. Beef extract peptone (PA), humic acid–vitamin (HV), YIM 91, and xylan–asparagine agar (XA), four media were used to isolate bacterial endophytes. The antibacterial and antifungal agents were 50 μg/mL cycloheximide, 25 μg/mL nalidixic acid, and 50 g/mL nystatin (Supplementary Table 1). Simultaneously, 0.2 mL of sterile water from the last washing of plant samples was coated on XA medium and incubated at 28°C for more than 3 weeks to elucidate the effect of plant surface disinfection. Single colonies were selected and inoculated into a purified culture medium using the four-zone scribing method and cultured at 28°C for 7 days.

The morphological characteristics of the strains were observed under an optical microscope. Representative strains were selected for molecular analysis.

### Identification of culturable endophytes and statistical analysis

Endophytic fungal attributions were identified via morphologic characteristics and ITS sequences. Genomic DNA was extracted using the CTAB method (Heinig et al., 2013). PCR was performed to amplify the internal transcriptional spacer of the fungi. ITS1 (5′-TCCGTAGGTGAACCTGCGG-3′) and ITS4 (5′-TCCTCCGCTTATTGATATGC-3′) were used as the universal primers (Qadri et al., 2014). BLAST was used to compare the sequences obtained after sequencing the amplification products with the sequences in GenBank; the related homology sequences were identified and downloaded. For the isolated endophytic bacteria, genomic DNA was extracted, and the 16S rDNA was amplified using PCR (Li et al., 2007). The universal primers used were 27f (5′-CAGAGTTTGATCCTGGCT-3′) and 1492r (5′-AGGAGGTGATCCAGCCGCA-3′) (Kim et al., 2012), and similarities were determined by comparing the sequences with the EzBioCloud database. The neighbor joining (NJ) method was used to construct the phylogenetic trees and repeated 1000 times using MegaX (Hall, 2013).

Relative frequency (RF) was used to estimate the taxa of specific endophytes from *S*. *dulcificum*, which was calculated as the number of isolates of one species divided by the total number of isolates (Wu et al., 2013). The diversity of the endophytes in the different plant tissues of *S*. *dulcificum* was estimated using three diversity indices. Shannon–Weiner index (*H′*) was calculated as $H^{\prime }=\sum_{i=1}^{k}p\,i\times \ln p\,i$, where *pi* = *Ni*/*N*, *Ni* is the number of isolates of an individual species of assemblage, and *N* is the total number of all species (Chao et al., 2005). The Shannon evenness (*E*) was calculated as *H′*/*H*_*max*_, where *H*_*max*_ = ln(*S*) and *S* is the total number of species in the sample. Simpson index (*D*) was used to determine the probability that two randomly selected individuals belong to the same species; the formula was as follows: *D* = 1 − ∑*pi*^2^, where *pi* = *Ni*/*N*, *Ni* is the number of isolates of the specific taxon of assemblage, and *N* is the total number of all species (Zhang et al., 2021). Sorensen’s (*QS*) and Jaccard’s (*JS*) indices of similarity were used to evaluate the similarity of the endophytic combinations among different tissues; the formulae were as follows: *QS* = 2*a*/(2*a* + *b* + *c*) and *JS* = *a*/(*a* + *b* + *c*), where *a* is the number of common species in both tissues and *b* and *c* are the number of species specific to the compared tissues (Osono and Mori, 2004).

### Fermentation and chemical extraction of the endophytes

To determine the effect of fermentation media on endogenous extracts, five liquid media were used for fungal fermentation: PDB, modified PDB (PDB^+^), MRS, malt extract (ME), and glucose peptone yeast (GPY). Meanwhile, four liquid media were used for bacterial fermentation: No. 61, No. 301, No. 312 and No. Z (Supplementary Table 1). Each seed culture was transferred into Erlenmeyer flasks containing different media and cultured at 28°C for 7 days on a rotary shaker at 200 rpm. The fermentation broth was mixed with ethyl acetate in a 1:1 ratio, extracted until the organic phase was nearly colorless, and concentrated to dryness under reduced pressure on a rotary evaporator. The obtained crude extracts were dissolved in methanol to determine biological activities.

### Antimicrobial assay of the endogenous extracts

Five indicator microorganisms, *Escherichia coli* (YM 3130), *Salmonella typhimurium* (YM 3115), *Candida albicans* (YM 2005), *Botrytis cinerea* (YM 3061), and *Fusarium solani* (YM 3158), were used to evaluate the antimicrobial activity of the extracts from the 88 isolates (68 fungal and 20 bacterial isolates) using the filter paper method. Before the antimicrobial activity assay, the pathogenic microorganism suspension was prepared. *E*. *coli* and *S. typhimurium* were cultured in LB medium at 37°C and 180 rpm for 12 h, whereas *C*. *albicans*, *B*. *cinerea*, and *F*. *solani* were cultured in PDB medium at 28°C and 180 rpm for 3 days. The prepared pathogenic microorganism suspension (200 μL) was absorbed using a pipette into a plate containing the corresponding medium and spread evenly with the applicator stick. The crude extracts were dissolved in methanol, and the filter paper sheets (diameter: 5 mm) were moistened with methanol and then placed on the plates. Each plate contained three samples, one positive control, and one negative control. Chloramphenicol (25 μg/mL) and nystatin (50 μg/mL) were used as the positive controls for the bacterial and fungal pathogens, respectively.

### Antioxidant assays of the endophyte extracts

#### Determination of DPPH radical scavenging capacity

The crude extracts (100 μL, 1 mg/mL) dissolved in methanol were mixed with DPPH solution (100 μL, 0.208 mM) in a 96-well microtiter plate and incubated at room temperature for 40 min in the dark. The absorbance was measured at 517 nm (Cheng et al., 2006). In this assay, pure methanol was used as the negative control, and V_*C*_ and V_*E*_ were the positive controls.


Radicalscavengingcapacityrates(%)=

$$\left[1-\left(A_{1}-A_{2}\right)/{\text{A}}_{0}\right]\times 100\%$$

where A_0_ is the absorbance of DPPH plus methanol, A_1_ is the absorbance of DPPH plus sample solution, and A_2_ is the absorbance of the sample solution plus methanol.

#### Determination of ABTS radical scavenging capacity

The ABTS working solution was prepared by mixing 5 mL of 7.4 mM ABTS radical solution with 88 μL of 2.6 mM potassium persulfate solution. The solution was incubated in the dark at 4°C for 12–16 h. Before the assay, the ABTS working solution was diluted with methanol to an absorbance of 0.7 ± 0.05. The crude extracts (100 μL, 1 mg/mL) were mixed with 1 mL of the ABTS solution and allowed to react for 10 min under dark conditions. Then, 200 μL of the sample was placed in a 96-well plate. The absorbance was measured at 734 nm using a microplate reader (Zhou et al., 2013). In this assay, pure methanol was used as the negative control, and V_*C*_ and V_*E*_ were the positive controls.


Radicalscavengingcapacityrates(%)=

$$\left[1-\left(A_{1}-A_{2}\right)/{\text{A}}_{0}\right]\times 100\%$$

where A_0_ is the absorbance of ABTS plus methanol, A_1_ is the absorbance of the ABTS plus sample solution, and A_2_ is the absorbance of the sample liquid plus methanol.

#### Yeast α-glucosidase inhibition assay of the endophyte extracts

Phosphate buffered saline (PBS, 0.1 M, pH 6.9) was used to prepare 1 mg/mL sample solution, 1 U/mL α-glucosidase, and 5 mM *p*-nitrophenyl-α-D-glucopyranoside (α-pNPG). The sample (30 μL) and α-glucosidase (50 μL) were mixed and reacted at 37°C for 10 min. Then 50 μL α-PNPG was added and incubated at 37°C for 20 min, and finally 100 μL Na_2_CO_3_ (0.1 M) was added and incubated at 37°C for 10 min to terminate the reaction. The absorbance was measured at 405 nm (Liao et al., 2022). The mixture without sample as the negative control, and acarbose as the positive control.


Inhibition(%)=[(A-B)-(C-D)]/(A-B)×100%

where A is the absorbance without sample solution, B is the absorbance without sample solution and enzyme, C is the absorbance with sample solution and enzyme, D is the absorbance without enzyme.

## Results

### Untargeted metabolomics analysis of different tissues

**FIGURE 2:**
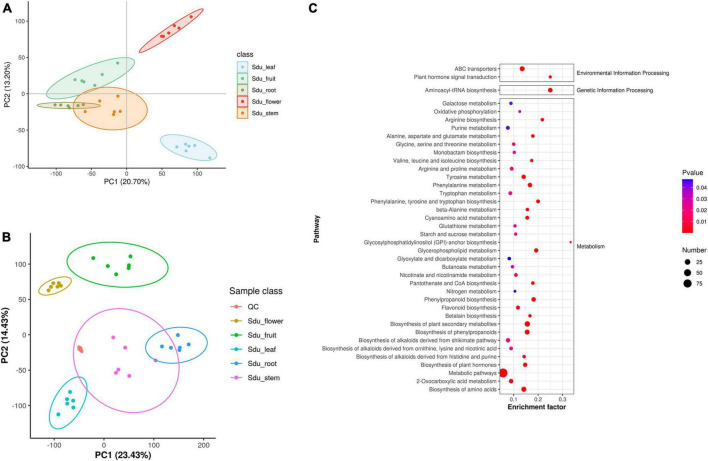
PLS-DA **(A)**, PCA of metabolome data **(B)** and KEGG pathway enriched dotplot **(C)**.

Untargeted metabolomics analysis was performed to elucidate the metabolic differences between the different tissues of *S*. *dulcificum* (Supplementary Figure 1). A total of 1,259 metabolites were annotated, including 377 lipids and lipid-like molecules, 217 organic acids and derivatives, 191 organoheterocyclic compounds, 155 benzenoids, 145 phenylpropanoids and polyketides, 111 organic oxygen compounds, 20 organic nitrogen compounds, 18 nucleosides, nucleotides, and analogues, 8 alkaloids and derivatives, 8 lignans, neolignans and related compounds, 7 organosulfur compounds, and 2 sulfadiazine (Supplementary Figure 2). The results of PLS-DA analysis indicated a strong correlation among the six biological replicates within the same tissue of *S*. *dulcificum*. Meanwhile, significant differences were observed in the composition of metabolites among different tissues, confirming the reliability of the analysis results (Figure 2A). Similarly, the principal component analysis (PCA) revealed that six biological duplicates of the same tissues can be clustered together, suggesting the reliability of the sequencing. Varying degrees of separation were observed between different tissues, indicating that the metabolites have obvious tissue specificity (Figure 2B). To investigate metabolite variations between different tissues, the five tissues were paired and the differences in metabolite content between the groups were compared. Metabolic analysis revealed that roots exhibited higher levels of tetrahydroisoquinolines, piperidines, sterol lipids and glycerolipids; stems showed higher levels of phenols, fatty acyls, keto acids and derivatives, cinnamic acids and derivatives; leaves had higher levels of cinnamic acids and derivatives, flavonoids, phenols, keto acids and derivatives, naphthalenes; flowers displayed higher levels of isoquinolines and derivatives, fatty acyls and furans; and fruits had higher levels of flavonoids, glycerolipids, phenols, diazines and purine nucleosides. Meanwhile, it was observed that flowers exhibited the highest metabolic activity among the tissues of *S*. *dulcificum*, while roots showed the lowest activity. Secondary metabolites in *S*. *dulcificum* were annotated utilizing the KEGG database. The annotated primary pathways encompass environmental information processing, genetic information processing, human diseases, and metabolism, with metabolism pathways representing the largest proportion (Figure 2C). The involved metabolic pathways included the biosynthesis of phenylpropanoids, biosynthesis of amino acids, ABC transporters, biosynthesis of plant hormones, 2-oxocarboxylic acid metabolism, biosynthesis of alkaloids and more.

### Diversity of uncultured endophytes

Supplementary Tables 2, 3 presents experimental data regarding the quality control of microbiomic high-throughput sequencing.

**FIGURE 3:**
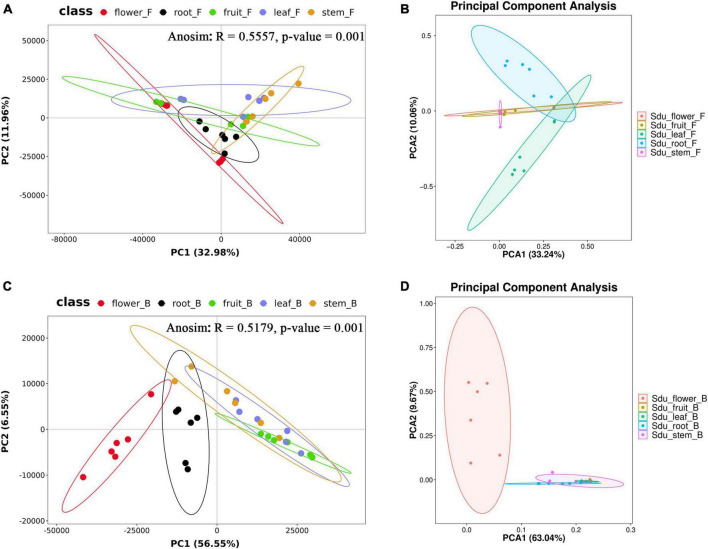
PLS-DA and PCA analysis of uncultured fungi **(A,B)** and bacteria **(C,D)** (the same group is displayed in ellipses with a 95% confidence interval).

For the fungal community, the mean Shannon index values for roots, stems, leaves, flowers, and fruits were 4.33, 3.30, 3.34, 2.03, and 2.05, whereas the mean Simpson index values were 0.85, 0.62, 0.72, 0.49, and 0.50, respectively. The results showed that the abundance and evenness of the fungal composition were the highest in roots; in contrast, no significant differences were observed between stems and leaves, and the lowest abundance and evenness were observed in flowers and fruits. Similarly, the Pielou-e index demonstrated that the fungi in roots had the most evenness in species composition, whereas flowers and fruits had the lowest evenness. Furthermore, Chao1 (roots, 257.67; stems, 259.50; leaves, 111.28; flowers, 118.00; and fruits, 72.42) and Observed species (roots, 257.33; stems, 259.50; leaves, 111.00; flowers, 117.83; fruits, 72.33) indices suggested that the fungal communities in roots and stems contained the largest number of species, followed by leaves and flowers; the lowest number of species was observed in fruits. Good’s coverage index was 1 for all tissue samples, indicating that the sequencing results represent the real situation of the samples (Table 1). PLS-DA and PCA analyses revealed that the endophytic fungal community composition of stems, flowers, and fruit are similar, with the highest differences in the fungal composition between roots and leaves. Additionally, Anosim analysis revealed a significant grouping effect with R = 0.556 and *P* = 0.001 (Figures 3A, B).

**TABLE 1:** Shannon, Simpson, Chao1, Goods coverage, Observed species and pielou-e index of uncultured fungi.

Sample	Chao1	Observed species	Good’s coverage	Shannon	Simpson	Pielou-e
root	257.6733 ± 120.90342b	257.33 ± 120.802b	1.00 ± 0	4.3317 ± 1.01991b	0.8467 ± 0.08779b	0.5450 ± 0.09524b
stem	259.5000 ± 130.92555b	259.50 ± 130.926b	1.00 ± 0	3.3017 ± 1.99190ab	0.6250 ± 0.33375ab	0.4017 ± 0.22230ab
leaf	111.2833 ± 66.91130a	111.00 ± 66.378a	1.00 ± 0	3.3383 ± 1.12734ab	0.7217 ± 0.14743ab	0.4967 ± 0.11483ab
flower	118.0050 ± 41.45171a	117.83 ± 41.369a	1.00 ± 0	2.0300 ± 0.38236a	0.4933 ± 0.1587a	0.2967 ± 0.03882a
fruit	72.4167 ± 22.81319a	72.33 ± 22.809a	1.00 ± 0	2.0500 ± 0.76511a	0.5017 ± 0.21958a	0.3367 ± 0.11708ab

*P*-values are based on matched samples *t*-test. Significant values (*P* < 0.05) are denoted by different small letters.

For the bacterial community, the Shannon index values were 3.95, 3.49, 2.24, 3.58, and 1.80 and Simpson index values were 0.85, 0.79, 0.61, 0.76, and 0.50 for roots, stems, leaves, flowers, and fruits, respectively; this suggests that the bacterial microbial composition exhibited the most abundance and evenness in roots. Although no significant difference was observed between stems and flowers, fruits had the lowest Pielou-e index (0.68, 0.63, 0.47, 0.51, and 0.42), indicating that the bacterial species composition exhibited the most evenness in roots and stems, followed by flowers; leaves and fruits had the lowest evenness in leaves and fruits. Furthermore, Chao1 (roots, 58.00; stems, 49.42; leaves, 27.06; flowers, 129.68; and fruits, 19.83) and Observed species (roots, 58.00; stems, 49.17; leaves, 27.00; flowers, 109.83; and fruits, 19.83) indices revealed that the number of bacterial community species in flowers was significantly higher than that in the other four tissues, followed by roots and stems. Leaves and fruits had the lowest number of species. The Goods coverage index was 1 for all tissue samples, suggesting that the sequencing results represent the real situation of the sample (Table 2). PLS-DA and PCA analyses suggested that the bacterial community composition in the flowers and roots of *S*. *dulcificum* significantly differ from that of the other three tissues, with the highest differences between roots and flowers and a more similar species composition in stems, leaves, and fruits. Furthermore, Anosim analysis revealed a significant grouping effect with R = 0.518 and *P* = 0.001 (Figures 3C, D).

**TABLE 2:** Shannon, Simpson, Chao1, Goods coverage, Observed species and pielou-e index of uncultured bacteria.

Sample	Chao1	Observed species	Good’s coverage	Shannon	Simpson	Pielou-e
root	58.0000 ± 19.51410b	58.00 ± 19.514a	1.00 ± 0	3.9500 ± .81056c	0.8533 ± 0.08116c	0.6783 ± 0.10647c
stem	49.4167 ± 30.36514ab	49.17 ± 30.459a	1.00 ± 0	3.4917 ± 1.17294bc	0.7867 ± 0.14137bc	0.6317 ± 0.12734bc
leaf	27.0633 ± 5.45995ab	27.00 ± 5.404a	1.00 ± 0	2.2383 ± 0.64561ab	0.6050 ± 0.15808ab	0.4683 ± 0.11990ab
flower	129.6767 ± 25.27427c	109.83 ± 59.172b	1.00 ± 0	3.5833 ± 1.28346bc	0.7617 ± 0.12922bc	0.5083 ± 0.16726abc
fruit	19.8333 ± 4.26224a	19.83 ± 4.262a	1.00 ± 0	1.8017 ± 0.56648a	0.5000 ± 0.14519a	0.4150 ± 0.10821a

*P*-values are based on matched samples *t*-test. Significant values (*P* < 0.05) are denoted by different small letters.

### Community composition and abundance of uncultured endophytes

**FIGURE 4:**
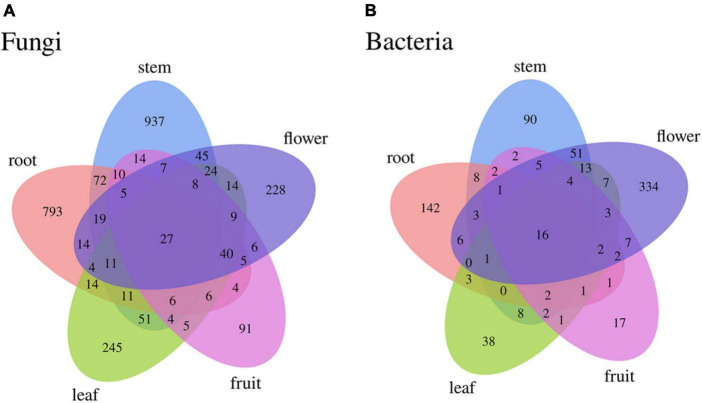
Venn diagram of uncultured endophytes identical and unique ASVs numbers statistics **(A)**: fungi, **(B)**: bacteria.

**FIGURE 5:**
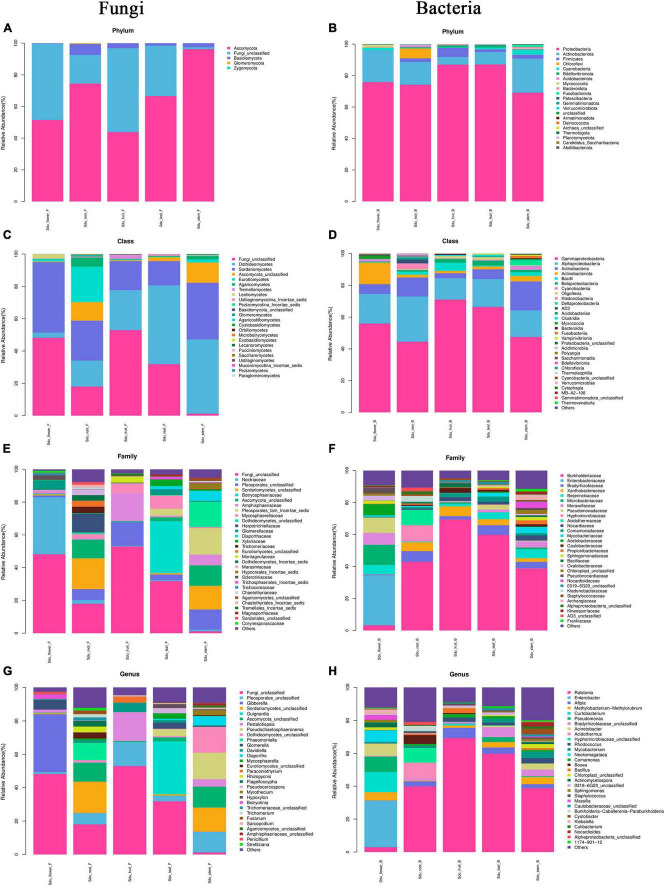
Mean relative abundance of the abundant phylum, class, family, and genus in uncultured fungi **(A,C,E,G)** and bacteria **(B,D,F,H)**.

Differences were observed in the endophytic groups among the different tissues of *S*. *dulcificum*. For the endophytic fungi, the numbers of 30 samples were calculated at the classification level of phylum, class, order, family, genus, and species, and 4913 ASVs were identified. The number of identical and unique ASVs in the different tissues was counted using a Venn diagram (Figure 4A), and the commonality and specificity of the fungal communities were analyzed. The number of identical ASVs was low at 27 for the five tissues, indicating a clear tissue specificity of the fungal composition of *S*. *dulcificum*. Furthermore, the number of stem-specific ASVs was the highest (937), with 793 for roots, 245 for leaves, 228 for flowers, and the lowest for fruits (91). By analyzing the fungal community structure at the phylum level, five phyla were obtained: Ascomycota, Basidiomycota, Glomeromycota, Zygomycota, and one unclassified unit (Figure 5A). Among them, Ascomycota and Basidiomycota were distributed in all five tissues, and Ascomycota accounted for > 40% of the species composition in all tissues and was the dominant phylum of the endophytic fungi in *S*. *dulcificum*. On the other hand, *Glomeromycota* and *Zygomycota* were only distributed in the roots. At the class level, 24 classes were obtained, with *Dothideomycetes*, *Sordariomycetes*, and *Eurotiomycetes* being the dominant groups of endophytic fungi (Figure 5C). At the family level, 170 families were obtained, with 13 families being distributed in all tissues (Figure 5E). In addition, at the genus level, 313 genera were obtained; *Gibberella*, *Guignardia*, *Pestalotiopsis*, *Glomerella*, *Davidiella*, *Diaporthe*, *Hypoxylon*, *Sarcopodium*, and *Penicillium* were identified in all tissues (Figure 5G). The dominant genera of roots, stems, leaves, flowers, and fruits were *Phaeomoniella* (10.51%), *Pseudochaetosphaeronema* (15.98%), *Guignardia* (31.72%), *Gibberella* (34.64%), and *Pestalotiopsis* (17.13%), respectively.

For the endophytic bacteria, the numbers of 30 samples were also calculated at the classification level of phylum, class, order, family, genus, and species, and 1703 ASVs were identified. Similarly, Venn diagrams were drawn to analyze the commonality and specificity of the bacterial communities (Figure 4B). The number of identical ASVs in the five tissues was small (16), suggesting that the bacterial composition of *S*. *dulcificum* exhibited obvious tissue specificity. The number of flower-specific ASVs (334) was significantly higher than that of the other four tissues, followed by roots (142) and stems (90); leaves and fruits had the lowest number of ASVs (38 and 17, respectively). By analyzing the bacterial community structure of *S*. *dulcificum* at the phylum level, 21 phyla were obtained; *Proteobacteria, Actinobacteria, Firmicutes, Cyanobacteria, Bdellovibrionota, and Bacteroidota* were distributed in all five tissues (Figure 5B). Proteobacteria accounted for 69.13%–87.10% of the species composition in the five different tissues and was the dominant phylum of endophytic bacteria, followed by *Actinobacteriota. Furthermore, Fusobacteriota* was only isolated from stems, *Verrucomicrobiota and Planctomycetota* were only isolated from roots, and *Thermotogota* was only isolated from fruits. At the class level, 47 classes were obtained; *Gammaproteobacteria, Alphaproteobacteria, Actinobacteria, and Bacilli* were the dominant taxa (Figure 5D). Six classes were only isolated from roots, whereas four classes were only isolated from stems; these results suggest that the potential for the isolation of specific bacteria from the roots and stems of *S*. *dulcificum* was higher than that from the other three tissues. At the family level, 145 families were obtained; 10 families were distributed in all tissues, and the taxa with the highest relative abundance in roots, stems, leaves, and fruits were *Burkholderiaceae*, with relative abundances of 42.92%, 38.80%, 59.82%, and 69.34%, respectively; in contrast, the dominant family in flowers was *Enterobacteriaceae* (31.25%) (Figure 5F). At the genus level, 232 genera were obtained, with *Ralstonia*, *Enterobacter*, *Afipia*, *Methylobacterium-Methylorubrum*, *Rhodococcus*, *Mycobacterium*, *Comamonas*, and *Cutibacterium* being present in all tissues (Figure 5H). Among roots, stems, leaves, and fruits, *Ralstonia* was the dominant genus, with relative abundances of 39.87%, 38.80%, 59.82%, and 69.34%, respectively. The dominant genus in flowers was *Enterobacter* (28.22%).

### Correlations between microorganisms and metabolites

To further understand the regulatory relationship between endophytes and secondary metabolites and their effects on different tissues, a multi-omics analysis was used to investigate the correlation between the microbiome and the metabolome. Considering the edible and medicinal value of *S*. *dulcificum* fruit, four comparison groups (fruit/flower, fruit/root, fruit/stem, fruit/leaf) were established to perform Spearman correlation analysis on inter-tissue differential metabolites and differential endophytes. In the comparisons between the fruit/flower and fruit/root groups, endophytic fungi showed only one and two significant correlations with plant metabolites, respectively, while bacteria exhibited 2,581 and 1,129 correlations with plant metabolites. On the contrary, in the comparisons between the fruit/stem and fruit/leaf groups, endophytic bacteria displayed 363 and 40 significant correlations with plant metabolites, whereas fungi exhibited 1,034 and 342 correlations with plant metabolites. Supplementary Table 4 presents the top 30 significant correlations for each comparison group; some comparison groups had less than 30 significant correlations.

Fungi belonging to the genera *Aspergillus*, *Asterotremella*, *Cyphellophora*, *Diaporthe*, *Dokmaia*, *Glomerella*, *Guignardia*, *Mycoleptodiscus*, *Ophioceras*, *Paraconiothyrium*, *Penicillium*, and *Xylaria*, as well as bacteria from the genera *Acidothermus*, *Acinetobacter*, *Actinomycetospora*, *Aureimonas*, *Corynebacterium*, *Enterobacter*, *Cryptosporangium*, *Jatrophihabitans*, *Labrys*, *P3OB-42*, *Burkholderia-Caballeronia-Paraburkholderia*, *Marmoricola*, *Methylobacterium*, *Micromonospora*, *Pseudomonas*, *Ralstonia*, *Rhodoplanes*, *Rhodopseudomonas*, *Sphingomonas*, and *Terriglobus*, exhibit significant correlations with the metabolite content of the host plant. Metabolites significantly correlated with the abundance of endophytes primarily include sterol lipids, prenol lipids, phenols, indoles and derivatives, flavonoids, fatty acyls, carboxylic acids and derivatives, and benzene and substituted derivatives. Of these, *Enterobacter* and *Guignardia* were significant positive correlated with the levels of quercetin-3-o-glucuronide, dihydrokaempferol, and zerumbone; *Rhodococcus* and *Sphingomonas* were significant negative correlated with the levels of epigallocatechin-3-gallate, (-)-epicatechin-3′-O-glucuronide, and arbutin. *Corynebacterium* was significant positive correlated with the levels of 3-o-p-coumaroylquinic acid and coumarin; however, it was significant negative correlated with 12-oxo-20-trihydroxy-leukotriene B4. Moreover, *Ralstonia* was significant negative correlated with the levels of (+)-usnic acid, 7,8,2′-trihydroxyflavone, and gallic acid 3-o-(6-galloylglucoside).

### Diversity and phylogenetics of culturable endophytes

Endophytes were isolated from healthy *S*. *dulcificum* tissues, and their diversity was determined.

**FIGURE 6:**
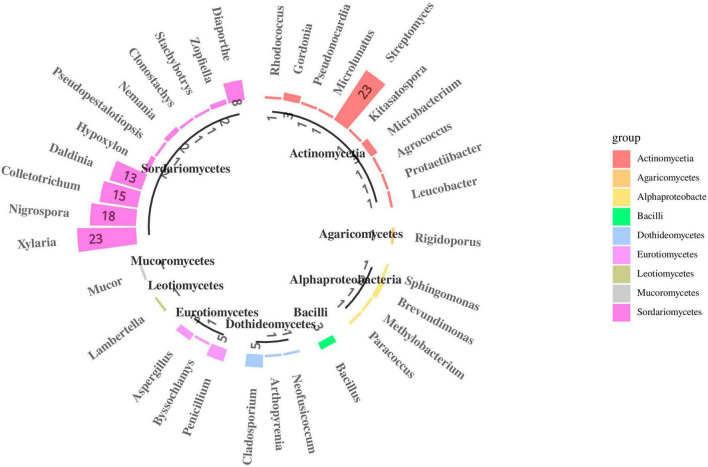
Species composition of endophytes from *S*. *dulcificum* (the number represents the count of isolated strains).

In total, 190 fungal isolates were obtained from 750 segments of five different tissues; 105 isolates were identified using ITS sequence and subsequent BLASTn comparison in the National Center for Biotechnology Information (NCBI) database. The ITS sequences of the representative fungi were deposited in GenBank; the accession numbers are listed in Supplementary Table 5. Phylogenetic reconstruction using the NJ algorithm revealed that the105 isolates could be classified into the phyla Ascomycota, Basidiomycota, and Mucoromycota (Figure 6). The isolated Ascomycetes included 18 genera, which belonged to Sordariomycetes (81.9%), Eurotiomycetes (8.6%), Dothideomycetes (6.7%), and Leotiomycetes (0.9%); The dominant fungal group *Sordariomycetes* mainly included *Xylaria* sp. (21.9%), *Nigrospora* sp. (17.1%), *Colletotrichum* sp. (14.3%), and *Daldinia* sp. (12.4%). This may be because these strains can rapidly sporulate and are easy to culture. Nine strains were isolated from the class *Eurotiomycetes* and belonged to three different genera: *Penicillium* sp., *Byssochlamys* sp., and *Aspergillus* sp. Seven strains were isolated from the class *Dothideomycetes*, which also belonged to three different genera: *Cladosporium* sp., *Arthopyrenia* sp., and *Neofusicoccum* sp. The least abundant classes were *Leotiomycetes*, which only contained strain S3 (*Lambertella* sp.). On the other hand, Basidiomycota included only one strain, i.e., S16 (*Rigidoporus* sp.), and Mucoromycota also included only one strain, i.e., L55 (*Mucor* sp.). Most isolates exhibited 98–100% homology with their close relatives.

Four different media were used to isolate endophytic fungi, and the maximum number of fungi was obtained using PDA (50.5%), followed by MRS (21.9%) and CYM (19.0%); the number of species was the lowest using CMA (8.6%) (Supplementary Figure 3A). Among the 20 genera, 13 exhibited high medium selectivity and were isolated from only one medium. The dominant strains *Xylaria* sp., *Nigrospora* sp., *Colletotrichum* sp., *Daldinia* sp., and *Phomopsis* sp. were isolated from all four media. *Rigidoporus* sp. and *Mucor* sp. were isolated from PDA and CYM, respectively. In terms of diversity, PDA and MRS were slightly higher than CMA and CYM. In general, PDA and MRS were the most suitable media for fungal isolation. Nevertheless, the number of strains isolated from the different plant tissues was different (Supplementary Figures 4A, C, E, G). The most abundant strains were isolated from leaves (34.3%), followed by stems (21.9%), flowers (16.2%), and fruits (15.2%); the lowest frequency was observed in roots (12.4%). This may be because endophytic fungi growth is affected by total phosphorus content, and the high phosphorus content in roots is not conducive to endophytic fungal colonization. In the leaves, *Xylaria* sp. exhibited the highest isolation frequency, followed by *Nigrospora* sp. and *Colletotrichum* sp. The endophytic fungi isolated only from the leaves were *Pseudopestalotiopsis* sp. and *Mucor* sp. In contrast, *Xylaria* sp. exhibited the highest isolation frequency in the stems, and *Clonostachys* sp., *Stachybotrys* sp., *Arthopyrenia* sp., *Lambertella* sp., and *Rigidoporus* sp. were only isolated from the stems. In flowers, *Colletotrichum* sp. and *Daldinia* sp. exhibited the highest isolation frequency; *Zopfiella* sp., *Byssochlamys* sp., and *Neofusicoccum* sp. were only isolated from the flowers. In fruits, *Nigrospora* sp. exhibited the highest isolation frequency, followed by *Phomopsis* sp. Furthermore, in roots, *Xylaria* sp., *Colletotrichum* sp., *Daldinia* sp., and *Aspergillus* sp. exhibited the highest isolation frequency.

Next, phylogenetic trees were constructed by selecting the ITS1–5.8S-ITS4 sequences of the adjacent fungi from GenBank (NCBI) (Supplementary Figure 5A). Five isolates, namely, FL21 (*Xylaria* sp.), FR27 (*Phomopsis* sp.), FR9 (*Phomopsis* sp.), FR36 (*Cladosporium* sp.), and S3 (*Lambertella* sp.), exhibited a sequence similarity of < 95% with known organisms in GenBank. These fungi may be potential new species. Furthermore, biodiversity analysis of the endophytic fungi revealed significant differences in the frequency of fungi obtained from the different tissues of *S. dulcificum*. The isolates from different tissues were compared based on their alpha diversity indices (Shannon–Wiener, Shannon evenness, and Simpson) and Sorensen’s and Jaccard’s indices of similarity to characterize the endophytic community (Supplementary Figures 6A, C). The Shannon–Wiener diversity (*H′*) revealed that the diversity of the fungal species in different tissues followed the order of stems (2.1802) > flowers (1.9554) > leaves (1.8925) > fruits (1.5837) > roots (1.5548). Slight differences in species evenness were observed between the five tissues. Furthermore, Simpson index (*D*) confirmed this result, with stems (0.8933) showing the highest diversity compared with the other tissues. Sorensen’s (*QS*) and Jaccard’s (*JS*) similarity values for the endophytic fungi from different tissues were 30%–80% and 18%–67%, respectively. Based on the similarity principle, the similarity coefficient of the endophytic fungi between the different tissues of *S. dulcificum* was between medium dissimilarity and medium similarity.

After more than 2 weeks of cultivation, 266 bacterial strains were isolated from the roots, stems, leaves, flowers and fruits of *S*. *dulcificum* samples. Subsequently, 46 representative strains were identified using molecular biological methods. The 16S rDNA sequences of the representative bacterial strains were deposited in GenBank; the accession numbers are listed in Supplementary Table 5. Phylogenetic reconstruction using the NJ algorithm revealed that they could be divided into the phyla Actinobacteria, Proteobacteria, and Firmicutes. The class Actinomycetia comprised 11 genera: *Mycolicibacterium* sp., *Rhodococcus* sp., *Gordonia* sp., *Pseudonocardia* sp., *Microlunatus* sp., *Streptomyces* sp., *Kitasatospora* sp., *Microbacterium* sp., *Agrococcus* sp., *Protaetiibacter* sp., and *Leucobacter* sp. Alphaproteobacteria contained four genera: *Sphingomonas* sp., *Brevundimonas* sp., *Methylobacterium* sp., and *Paracoccus* sp. The class Bacilli contained only one genus: *Bacillus* sp. (Figure 6). The dominant bacterial group isolated was Actinomycetia, accounting for 80.4% of all strains; this may be because more isolation media suitable for Actinomycetia growth and propagation were selected in the present study. Among them, 23 strains were identified as the genus *Streptomyces*. Only one strain was isolated from each of the 10 genera: *Protaetiibacter* sp., *Leucobacter* sp., *Kitasatospora* sp., *Agrococcus* sp., *Pseudonocardia* sp., *Microlunatus* sp., *Sphingomonas* sp., *Rhodococcus* sp., *Paracoccus* sp., and *Methylobacterium* sp. Nevertheless, few rare strains were isolated in this study. Most isolates shared 98%–100% homology with their close relatives.

Diversity analysis of the endophytic bacteria isolated from four different media revealed that the number of Actinomycetia isolated from XA was the highest (39.1%), with relatively rich species diversity (Supplementary Figure 3B). We hypothesized that the xylan and amino acids in XA can provide better nutrition for the growth and reproduction of endophytic actinomycetes. Furthermore, no significant differences were observed in the number of endophytic bacteria isolated from HV, PA, and YIM 91; *Streptomyces* sp. was isolated from these three media. Alphaproteobacteria and Bacilli strains were only isolated from PA. Among the 16 genera, 12 exhibited high medium selectivity and were only isolated from one medium. The species and isolation frequencies of the endophytic bacteria in different tissues were different (Supplementary Figures 4B, D, F, H). The highest number of bacteria was isolated from flowers, whereas the lowest number of bacteria was isolated from leaves (flowers > fruits > stems > roots > leaves). Twenty-two endophytic bacterial strains were isolated from flowers and distributed in four genera. *Streptomyces* sp. had the highest isolation frequency, and the other three genera were *Microbacterium* sp., *Bacillus* sp., and *Brevundimonas* sp. The pure culture strains isolated from fruits had the highest diversity, and a total of seven genera were isolated: *Streptomyces* sp. (two strains), *Protaetiibacter* sp., *Agrococcus* sp., *Pseudonocardia* sp., *Sphingomonas* sp., *Methylobacterium* sp., and *Brevundimonas* sp. Among them, the genera *Protaetiibacter*, *Agrococcus*, *Sphingomonas*, *Methylobacterium*, and *Pseudonocardiawere* were only isolated from the fruits. We hypothesize that *S*. *dulcificum* has more abundant actinomycetes resources in fruits than in other tissues. Five genera of endophytic bacteria were isolated from roots: *Mycolicibacterium* sp., *Microbacterium* sp., *Streptomyces* sp., *Kitasatospora* sp., and *Leucobacter* sp.; of them, three genera, namely, *Mycolicibacterium* sp., *Kitasatospora* sp., and *Leucobacter* sp., were specific to roots. Five genera of bacteria, namely, *Streptomyces* sp., *Microbacterium* sp., *Gordonia* sp., *Rhodococcus* sp., and *Bacillus* sp., were isolated from the stems of *S*. *dulcificum*; *Rhodococcus* sp. was the stem-specific genus. Only three strains were isolated from the leaves: *Paracoccus* sp., *Gordonia* sp., and *Microlunatus* sp. Among them, *Paracoccus* sp. and *Microlunatus* sp. were the genera specifically isolated from the leaves of *S*. *dulcificum*.

Subsequently, the 16S rDNA sequences of adjacent bacteria were selected from the EzBioCloud database to construct the phylogenetic trees (Supplementary Figure 5B). After cloning and sequencing, four Actinomycetia strains were identified as potential novel taxonomic units; their similar strains and similarities were *Protaetiibacter intestinal* (fr5, similarity: 98.41%), *Paenalcaligenes suwonensis* (s4, similarity: 98.2%), *Gordonia didemni* (s8, similarity: 98.11%), and *Streptomyces avicennia* (fl7, similarity: 97.69%). Diversity analysis revealed significant differences in the frequency of endophytic bacteria obtained from different tissues. The strains isolated from the different tissues were compared based on their alpha diversity indices (Shannon–Wiener index, Shannon evenness, and Simpson’s diversity index) and Sorensen’s (*QS*) and Jaccard’s (*JS*) indices of similarity to characterize the endophytic bacterial community (Supplementary Figures 6B, D). Shannon–Wiener diversity (*H′*) revealed that the diversity of bacteria in the different tissues was fruits (1.9387) > roots (1.5699) > stems (1.5466) > leaves (1.0989) > flowers (0.6566). Simpson index (*D*) revealed the following: leaves (1.000) > fruits (0.9643) > roots (0.9333) > stems (0.9048) > flowers (0.3333). Sorensen’s and Jaccard’s similarity revealed that the endophytic bacteria ranged between 0–67% and 0–50%, respectively, indicating the low similarity of the endophytic bacteria from different tissues.

### Comparison of the diversity of uncultured and culturable endophytes

At the genus level, the community compositions of uncultured and culturable endophytes in the different tissues of *S*. *dulcificum* were compared. Among the 313 fungal genera analyzed via high-throughput sequencing, 11 genera could be cultured and isolated, whereas nine genera could be cultured and isolated but not analyzed via high-throughput sequencing. Supplementary Figure 7A illustrates the comparison of the uncultured and culturable endophytic fungi in the different tissues. For the bacteria, 10 of the 232 genera analyzed via high-throughput sequencing were isolated in culture, and six culturable genera were not analyzed via high-throughput sequencing. Supplementary Figure 7B illustrates the comparison of the uncultured and culturable bacteria in different tissues. High-throughput sequencing and culture isolation revealed that the same strains were not identified in the roots and leaves. The culturable strains isolated from flowers were all analyzed via high-throughput sequencing.

### Antimicrobial activity of the endogenous extracts

The 68 fungal strains and 20 bacterial strains were inoculated in PDB and YIM 91 media, respectively, and cultured at 28°C for 3 days to obtain the seed liquid. The endogenous extracts from *S. dulcificum* were subjected to antimicrobial activity measurements using the filter paper method against five pathogenic microorganisms.

**FIGURE 7:**
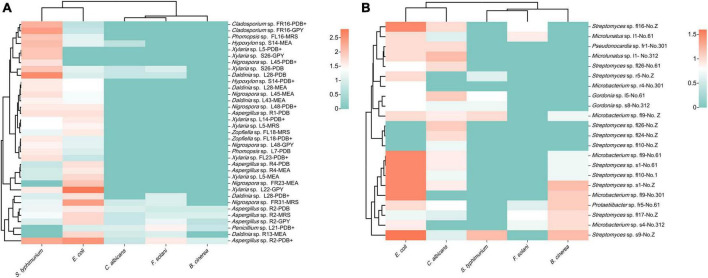
Heatmap of antimicrobial activity of strain extracts against pathogenic microorganisms **(A)**: fungi; **(B)**: bacteria (different colors represent varying sizes of inhibition circles, with a deeper red hue indicating a larger diameter of the inhibition circle).

Seventeen fungal strains possessed broad-spectrum antimicrobial activity and could inhibit both fungal and bacterial pathogensz (Figure 7A). In addition, 37 fungal strains were only active against bacterial pathogens, eight strains were only active against fungal pathogens, and six strains were inactive against all pathogens. Fermentation crude extracts from different media of the same strain revealed different activities against the same pathogen. The extracts of strains FR23 (*Nigrospora* sp., MEB), FR31 (*Nigrospora* sp., MRS), L22 (*Xylaria* sp., GPY), and R2 (*Aspergillus* sp., PDB) exhibited strong inhibitory activities against *E. coli*. The inhibition circle sizes were 2.0, 2.4, 2.8, and 2.5 cm, respectively. On the other hand, the extracts of strains L28 (*Daldinia* sp., PDB), S26 (*Xylaria* sp., PDB and GPY), FL16 (*Phomopsis* sp., MRS and GPY), FR23 (*Nigrospora* sp., MEB), FR16 (*Cladosporium* sp., PDB^+^ and GPY), and R2 (*Aspergillus* sp., PDB) exhibited significant inhibition against *S. typhimurium* (inhibition circle sizes of 2.6, 2.0, 2.0, 2.0, 2.0, 2.2, 2.4, 2.0, and 2.3 cm, respectively). The extracts of strain L21 (*Xylaria* sp., PDB^+^) and R2 (*Aspergillus* sp., PDB) exhibited significant inhibition against *F. solani* (inhibition circle sizes of 1.5 and 1.6 cm, respectively). The fungi that exhibited strong inhibitory activity against pathogenic microorganisms mainly belonged to Sordariomycetes and Eurotiomycetes.

The inhibition experiments of endophytic bacteria revealed that 16 strains exhibited different degrees of antimicrobial activity (Figure 7B). Among them, the extracts of strains s1 (*Streptomyces* sp., No. 61 and Z), s9 (*Streptomyces* sp., No. Z), fl9 (*Microbacterium* sp., No. 61 and 301), fl16 (*Streptomyces* sp., No. Z), and fl10 (*Streptomyces* sp., No. 61) exhibited obvious inhibitory effects against *E. coli*. The inhibition circle sizes were 1.5, 1.5, 1.6, 1.5, 1.5, 1.5, and 1.5 cm, respectively. *Streptomyces* primarily contributed to the antimicrobial activity of endophytic bacteria. Among the five pathogenic microorganisms selected, the endophytic bacteria of *S. dulcificum* exhibited more pronounced inhibition against *E. coli* and weaker inhibition against *F. solani* and *S. typhimurium*.

### Antioxidant activity of the endogenous extracts

The endogenous extracts from *S. dulcificum* were subjected to antioxidant activity measurements using the DPPH and ABTS methods.

**FIGURE 8:**
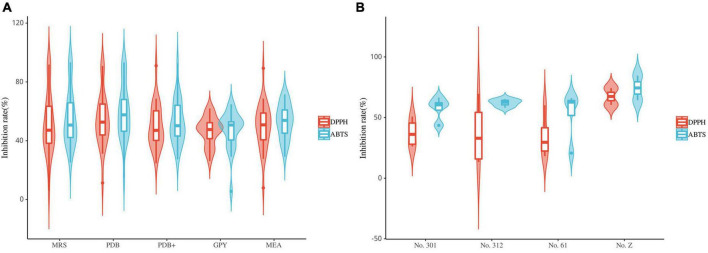
Violin plot of antioxidant activity of endophyte extracts **(A)**: fungi; **(B)**: bacteria (the horizontal line inside the box represents the median of free radical scavenging rate; the upper and lower edges of the box represent the upper and lower quartiles; the top and bottom of the vertical line represent the maximum and minimum values).

Most fungal strains exhibited strong or weak antioxidant activity (Figure 8A). Furthermore, these fungal extracts exhibited different radical scavenging capabilities for DPPH and ABTS radicals. The radical scavenging capabilities of most fungal extracts were 40%–70%. Among them, the extracts of strains S9 (*Xylaria* sp., MRS), L5 (*Xylaria* sp., MRS, PDB, and PDB^+^), L52 (*Xylaria* sp., PDB), FL23 (*Xylaria* sp., MEB), S11 (*Nemania* sp., MRS and PDB) and FL43 (*Daldinia* sp., MRS) exhibited strong antioxidant activity. These extracts exhibited radical scavenging capacities comparable to the positive control. This result suggests that the isolates classified as Sordariomycetes primarily contribute to the observed radical scavenging capability. In addition, extracts from different media of the same strain exhibited different radical scavenging capabilities. The extracts that exhibited strong radical-scavenging capability were mostly from MRS and PDB, while extracts from GPY exhibited relatively poor antioxidant activity. All extracts of the 29 fungal strains did not exhibit radical scavenging capabilities in both DPPH and ABTS assays.

The results of the DPPH and ABTS radical scavenging assays suggest that the antioxidant activity of the endophytic bacterial extracts of *S. dulcificum* is weak (Figure 8B). Ten endophytic bacterial strains exhibited antioxidant activity. Among them, only one endophytic bacteria strain (s9, *Streptomyces* sp., No. Z) exhibited strong ABTS radical scavenging capacity. The radical scavenging capacities of these bacterial extracts varied: the same extracts exhibited significantly different activities in the DPPH and ABTS assays. In addition, extracts from different media of the same strain had different antioxidant activities. These strains exhibiting antioxidant activity belonged to Actinomycetia, except fr10, which belonged to the genus *Brevundimonas*.

### α-glucosidase inhibition activity of the endophytic fungal extracts

**FIGURE 9:**
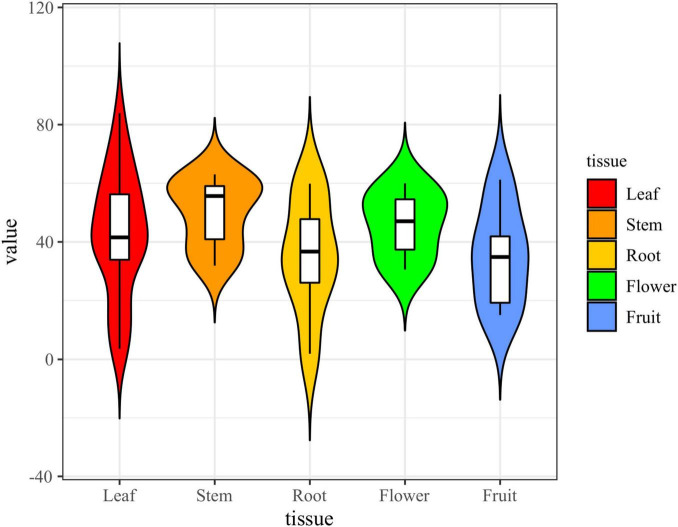
Violin plot of α-glucosidase inhibitory activity of fungal extracts (the black horizontal line inside the box represents the median of α-glucosidase inhibition rate; the upper and lower edges of the white box indicate the upper and lower quartiles; the top and bottom ends of the vertical line represent the maximum and minimum values).

The results showed that the crude extracts of 58 (85.29%) fungal strains exhibited strong or weak inhibitory activity, and most of the extracts inhibition of α-glucosidase was concentrated in the range of 35%-60% (Figure 9). The inhibition rate of acarbose on α-glucosidase reached 94.44%, and the inhibition rate of 23 strains of crude extract was higher than 50% under the same concentration. The strains that showed strong inhibitory activity were assigned to the genera *Xylaria* (six strains), *Colletotrichum* (three strains), *Nigrospora* (three strains), *Daldinia* (one strains), *Mucor* (one strains), *Penicillium* (one strains), *Hypoxylon* (one strains), *Stachybotrys* (one strains), *Cladosporium* (two strains), *Aspergillus* (two strains), *Neofusicoccum* (one strains) and *Phomopsis* (one strains). Among them, the extract of strain L52 (*Xylaria* sp.) showed the strongest α-glucosidase inhibitory activity (83.97%). Among the 23 fungal isolates, 10 were obtained from leaves, six from stems, two from roots, three from flowers, and two from fruits. Overall, endophytic fungi isolated from stems exhibited higher inhibitory activity than those from other tissues, followed by flowers, while those from roots and fruits showed the lowest inhibitory activity.

## Discussion

Xishuangbanna Prefecture of Yunnan Province is located at 21°8′–22°36′ north latitude and 99°56′–101°51′ east longitude, with a typical monsoon climate (Yang et al., 2008). Due to its unique climatic and geographical conditions, this region hosts a diverse and complex biota with high species diversity. *S*. *dulcificum* was cultivated in the Yunnan Provincial Institute of Tropical Crop Science, located in Xishuangbanna Prefecture, and then introduced in 1964 when Premier Zhou Enlai visited Ghana. The plant has edible, medicinal, and ornamental value. At present, studies on *S. dulcificum* primarily focus on the composition and pharmacological activity of its plant extracts, with a knowledge gap on its endophytic resources. Microorganisms colonized in plants have important effects on plant growth, health, and secondary metabolism. Simultaneously, the plant serves as a living environment for the growth and reproduction of numerous endophytes (Trivedi et al., 2020). More than 150 genera of endophytic fungi have been isolated from plants in temperate, tropical, and subtropical regions (Ghanta et al., 2012; Bernard et al., 2017). The phyla Ascomycota and Basidiomycota are the most common endophytic fungi isolated from plants (Vijayaraghavan and Yun, 2008). Additionally, bacteria constitute an important community of plant endophytes, showcasing complex species diversity. Actinobacteria, Proteobacteria, Firmicutes, and Bacteroidetes are the dominant bacterial phyla in plant endophytes (Trivedi et al., 2020). These findings are consistent with those of the present study. However, information on plant endophytic resources remains scarce because the laboratory cannot fully simulate the natural growth environment of endophytes.

In the present study, we reported the diversity and bioactivities of the endophytes in *S. dulcificum* for the first time. High-throughput sequencing was performed to elucidate the diversity and community structure of endophytes in different tissues of *S. dulcificum*. For endophytic fungi, 1031, 1251, 479, 466, and 247 ASVs were identified in roots, stems, leaves, flowers, and fruits, respectively. In addition, only 27 identical ASVs were observed in the five tissues; the unique ASVs in roots (793) and stems (937) were higher than those in leaves (245), flowers (228), and fruits (91). For bacteria, 190, 208, 101, 455, and 68 ASVs were identified in roots, stems, leaves, flowers, and fruits, respectively. Only 16 identical ASVs were observed in the five tissues; the number of unique ASVs in flowers (334) was significantly higher than that in roots (142), stems (90), leaves (38), and fruits (17). At the phylum level, Ascomycota and Proteobacteria were the dominant taxa in *S*. *dulcificum*. In addition, the dominant families and genera of endophytic bacteria in roots, stems, leaves, and fruits were consistent, whereas the dominant groups in flowers differed from those in the other four tissues. Unlike fungi, the number of endophytic bacteria in flowers was significantly higher than in the other tissues. However, both endophytic bacteria and fungi exhibited the lowest ASVs in fruits. Further studies are to be focused on exploring the reasons for these differences.

In this study, we noted significant differences in the metabolomes of the different tissues of *S. dulcificum*. The fruits of *S. dulcificum*, known for their high edibility and potential medicinal value, have become a focal point in current research. Previous studies have reported that fruits contain more phenols and flavonoids, which are ideal antioxidants (Swamy et al., 2014). Furthermore, the research has indicated the antidiabetic effect of pulp extract and the cholesterol-lowering activity of triterpenoids in seed extract (Han et al., 2019; Huang et al., 2020). Our findings revealed higher flavonoid content in fruits and leaves, while phenol content was elevated in fruits, stems, and leaves. These results suggested that the extracts of *S. dulcificum* leaves are equally worthy of further study for identifying natural antioxidants. Next, Spearman’s correlation analysis revealed significant correlations between endophytic bacteria and plant metabolites in the fruit/flower and fruit/root groups. Additionally, a notable correlation was observed between endophytic fungi and plant metabolites in the fruit/stem and fruit/leaf groups. Taken together, these results suggested obvious differences in the effects of endophytic fungi and bacteria on the plant metabolites in various tissues of *S. dulcificum*. The reasons for such differences are worthy of further investigation.

Additionally, 190 fungal strains were isolated from 750 fragments of the different tissues of *S. dulcificum*, and 105 strains were further identified via molecular biology methods. These belonged to 20 genera, suggesting the great diversity of the endophytic fungi in *S. dulcificum*. *Sordariomycetes* dominated the endophytic fungal community, constituting 81.9% of the total isolates, followed by Eurotiomycetes and Dothideomycetes. Notably, *Xylaria*, *Nigrospora*, *Colletotrichum*, and *Daldinia* were the dominant genera. Moreover, the diversity index revealed differences in the diversity of endophytic fungal communities among the five tissues, with high diversity in stems, flowers, and leaves, and comparatively lower diversity in fruits and roots. Among the 20 fungal genera, 14 displayed tissue specificity, likely attributed to the species specificity of endophytes and distinct ecological conditions in different environments. A total of 266 endophytic bacterial strains were isolated from diverse tissues of *S. dulcificum* using the coating method, with molecular biology techniques identifying 46 strains. These bacterial strains were classified into three phyla, three classes, and 16 genera. Actinomycetia represented the dominant group, comprising 80.4% of the total species, with *Streptomyces* as the dominant genus (50%). The diversity index revealed significant differences in the diversity of endophytic bacterial communities in the five tissues, with the highest diversity in fruits and the lowest in flowers. Interestingly, the colonization rate of endophytic bacteria in flowers was the highest. Eleven of the 16 genera of endophytic bacteria exhibited tissue specificity, suggesting the high tissue specificity of endophytic bacteria in *S. dulcificum*. In our upcoming experiments, we plan to incorporate confocal microscopy imaging of isolated microbes inside the plant tissues to confirm their endophytic nature.

By comparing the results of high-throughput sequencing with the isolation of culturable endophytes, we observed both similarities and differences in the abundance and diversity of uncultured and culturable endophytes in different tissues of *S. dulcificum*. The abundance and diversity of uncultured and culturable endophytic fungi obtained from stems were higher than those from the other four tissues, with flowers having the highest number of both culturable and uncultured endophytic bacterial species. In contrast, roots exhibited higher abundance and diversity of uncultured endophytic fungi but the lowest level of culturable endophytic fungi. The number of uncultured endophytic fungal species was highest in stems, whereas culturable endophytic fungi were most abundant in leaves. Among the 313 fungal genera and 232 bacterial genera analyzed via high-throughput sequencing, 11 fungal genera and 10 bacterial genera were isolated by pure culture method. Fungi included the genera *Xylaria*, *Nigrospora*, *Daldinia*, *Diaporthe*, *Penicillium*, *Nemania*, *Hypoxylon*, *Aspergillus*, *Stachybotrys*, *Lambertella and Cladosporium*; bacteria include the genera *Streptomyces*, *Pseudonocardia*, *Microbacterium*, *Microlunatus*, *Rhodococcus*, *Brevundimonas*, *Paracoccus*, *Sphingomonas*, *Methylobacterium* and *Bacillus*. The number and species of endophytes analyzed via high-throughput sequencing were much higher than the number of strains that could be isolated in culture. The reason for this discrepancy may be the limitations of microbial culture techniques and the growth conditions of endophytes, many of which are difficult to artificially cultivate. The combination of various basic and selective media can considerably improve the isolation and culture efficiency of endophytes. In addition, the fungal ASVs analyzed via high-throughput sequencing were much higher than the bacterial ASVs. We will further investigate whether the reason for this phenomenon is the limitations of sequencing technology and databases or the species characteristics of *S. dulcificum*.

*In vitro* experiments have clarified the antimicrobial, antioxidant and hypoglycemic abilities of different medium extracts of the endophytes, enhancing our understanding of the symbiotic relationship between host plants and endophytes. In this study, 68 fungal and 20 bacterial strains were screened for their antimicrobial and antioxidant activities. Among the 68 fungal strains, 91.2% exhibited antimicrobial activity, and 57.4% exhibited free radical scavenging capacity. In contrast, among the 20 bacterial strains, 80% exhibited antimicrobial activity and 50% exhibited weak free radical scavenging capacity. Actinobacteria, Sordariomycetes, and Eurotiomycetes were the primary contributors to antimicrobial activity. In addition, 58 fungal strains exhibited α-glucosidase inhibitory activity, with inhibition concentrations ranging from 35–60%. Fungi of the genera *Xylaria*, *Colletotrichum*, *Cladosporium* and *Aspergillus* showed strong inhibitory activity, with strain L52, a fungus of the genus *Xylaria*, demonstrating the highest inhibitory activity at 83.97%. These results suggested that endophytic fungi are the primary source of active endophytes, providing valuable insights for future research. Subsequent studies in our laboratory will primarily focus on endophytic fungi, with consideration given to the potential novelty of five fungal strains and four bacterial strains. These strains, as well as those exhibiting strong antimicrobial, antioxidant, or hypoglycemic activity, will be considered for further chemical screening for secondary metabolite-related studies to isolate novel bioactive secondary metabolites. These new secondary metabolites can be potentially novel natural antimicrobial, antioxidant and hypoglycemic drugs. Furthermore, the subsequent research would be enhanced by conducting additional plant experiments to validate the observed *in vitro* biological activities.

In conclusion, the endophytes in *S. dulcificum* from Xishuangbanna exhibit abundant diversity, with some strains displaying remarkable bioactivity. Our study results suggest that the endophytes obtained from *S. dulcificum* have the potential to produce novel active secondary metabolites, providing valuable resources for the development of biopesticides and antibiotics. Currently, the ongoing studies on the secondary metabolites of the endophytes obtained from *S. dulcificum* are being conducted.

## Data availability statement

The datasets presented in this study can be found in online repositories. The names of the repository/repositories and accession number(s) can be found below: https://www.ncbi.nlm.nih.gov/, PRJNA975717 and PRJNA975836.

## Author contributions

SL: Conceptualization, Methodology, Software, Writing− original draft. YH: Methodology, Writing−review and editing. KZ: Methodology, Writing−review and editing. QM: Methodology, Writing−review and editing. MW: Software, Writing−review and editing. SS: Resources, Supervision. Writing−review and editing. SW: Conceptualization, Methodology, Writing−review and editing.
