# Supplementary material for: Exploring the diversity, bioactivity of endophytes, and metabolome in Synsepalum dulcificum
Source: Front Microbiol. 2024 Feb 27;15:1258208. doi: 10.3389/fmicb.2024.1258208 (PMC10929569; doi:10.3389/fmicb.2024.1258208)
Supplement: Supplementary file 1 [file Data_Sheet_1.docx]

Supplementary Tables

**Tables legend**

**TABLE S1** Eight different media for endophyte isolation and nine for fermentation

**TABLE S2** Sequencing statistics data of fungal samples

**TABLE S3** Sequencing statistics data of bacterial samples

**TABLE S4** The top 30 significant correlations for each comparison group

**TABLE S5** GenBank accession number of 41 fungi and 28 bacterial

Supplementary Figures

**Figures legend**

**FIGURE S1** Total ion chromatogram and metabolite m/z-rt distribution map

**FIGURE S2** The pie figure of classification of all metabolites detected in 5 tissues from *Synsepalum* *dulcificum*.

**FIGURE S3** Venn diagram of endophytes isolated from different media (**A**: fungi, **B**: bacteria).

**FIGURE S4** Mean relative abundance of the abundant phylum, class, family, and genus in culturable fungi **(A, C, E, G)** and bacteria **(B, D, F, H)**.

**FIGURE S5** Phylogenetic tree of partial sequence of endophytes from *S*. *dulcificum* (**A**: fungi; **B**: bacteria).

**FIGURE S6** Alpha diversity, Sorensen’s and Jaccard’s index of similarity of culturable fungi **(A, C)** and bacteria **(B, D)**.

**FIGURE S7** Comparison of culture-free uncultured and culturable strains in different tissues (**A**: fungi; **B**: bacteria).

**TABLE S1** Eight different media for endophyte isolation and nine for fermentation

| Medium | Ingredient |
| --- | --- |
| PDA | 200 g potato, 20 g glucose, 15 g agar, 1000 ml sterile water |
| MRS | 5 g tryptone, 1 g K_2_HPO_4_, 2 g yeast, 0.5 g MgSO_4_, 20 g glucose, 15 g agar, 1000 ml sterile water |
| CMA | 20 g corn meal, 15 g agar, 1000 ml sterile water |
| CYM | 20 g glucose, 2 g peptone, 2 g yeast extract powder, 0.5 g MgSO_4_, 1 g K_2_HPO_4_, 15 g agar, 1000 ml sterile water |
| HV | 1.0 g humic acid, 2.64 g (NH_4_)_2_SO_4_, 2 g NaCl, 2 g KCl, 2 g MgCl_2_·6H_2_O, 1 g K_2_HPO_4_, 0.2 g KNO_3_, 0.2 g CaCO_3_, 0.01 g FeSO_4_, 3.7 mg multivitamin, 15 g agar, 1000 ml sterile water |
| PA | 10 g tryptone, 5 g beef paste, 5 g NaCl, 15 g agar, 1000 ml sterile water |
| YIM 91 | 0.1 g proteose peptone, 1 g cysteine, 1 g trehalose, 4 g NaHCO_3_, 0.45 g K_2_HPO_4_, 0.9 g NaCl, 0.09 g MgSO_4_·7H_2_O, 0.09 g CaCl_2_, 10 mg chlorhematin, 10 μg biotin, 15 g agar, 1000 ml sterile water |
| XA | 2.5 g xylan, 1g asparaginate, 0.5 g K_2_HPO_4_, 0.25g KNO_3_, 0.2 g MgSO_4_·7H_2_O, 0.01 g FeSO_4_, 0.5 g CaCl_2_, 15 g agar, 1000 ml sterile water |
| PDB | 200 g potato, 20 g glucose, 1000 ml sterile water |
| PDB^+^ | 200 g potato, 20 g glucose, 3 g MgSO_4_, 10 mg V_B_, 1000 ml sterile water |
| MRS | 20 g glucose, 5 g tryptone, 1 g K_2_HPO_4_, 2 g yeast extract, 0.5 g MgSO_4_, 1000 ml sterile water |
| MEB | 30 g malt extract, 3 g soy peptone, 1000 ml sterile water |
| GPY | 10 g glucose, 3 g peptone, 7 g yeast extract, 1 g KH_2_PO_4_, 0.5 g MgSO_4_, 1000 ml sterile water |
| No. 61 | 20 g soybean meal, 2 g peptone, 20 g glucose, 5 g soluble starch, 4 g NaCl, 2 g yeast extract, 0.5 g K_2_HPO_4_, 0.5 g MgSO_4_·7H_2_O, 2 g CaCO_3_, 1000 ml sterile water |
| No.301 | 24 g soluble starch, 3 g beef extract, 1 g glucose, 5 g yeast extract, 3 g peptone, 4 g CaCO_3_, 1000 ml sterile water |
| No. 312 | 10 g glucose, 10 g glycerin, 2.5 g corn flour, 5 g peptone, 10 g soluble starch, 2 g yeast extract, 3 g CaCO_3_, 1 g NaCl, 1000 ml sterile water |
| No. Z | 25 g soluble starch, 15 g soybean meal, 2 g yeast extract, 4 g CaCO_3_, 1000 ml sterile water |

**TABLE S2** Sequencing statistics data of fungal samples

| Sample | Raw Tags | Raw Bases (M) | Valid Tags | Valid Bases (M) | Valid% | Q20% | Q30% | GC% |
| --- | --- | --- | --- | --- | --- | --- | --- | --- |
| Sdu_root_F1 | 82013 | 40.44 | 66977 | 18.11 | 94.73 | 99.46 | 98.16 | 57.39 |
| Sdu_root_F2 | 87838 | 43.79 | 83028 | 22.35 | 92.64 | 99.48 | 98.25 | 54.98 |
| Sdu_root_F3 | 84937 | 40.84 | 80971 | 22.36 | 95.25 | 99.45 | 98.12 | 55.44 |
| Sdu_root_F4 | 85043 | 42.47 | 81077 | 21.39 | 95.33 | 99.56 | 98.45 | 54.23 |
| Sdu_root_F5 | 80366 | 43.92 | 72620 | 19.00 | 94.52 | 99.45 | 98.06 | 54.64 |
| Sdu_root_F6 | 86472 | 41.01 | 82349 | 23.48 | 81.67 | 99.38 | 97.94 | 58.17 |
| Sdu_stem_F1 | 86260 | 40.10 | 82379 | 21.62 | 93.70 | 99.61 | 98.60 | 58.32 |
| Sdu_stem_F2 | 83660 | 42.38 | 75734 | 19.38 | 94.47 | 99.28 | 97.66 | 56.45 |
| Sdu_stem_F3 | 80805 | 42.08 | 76938 | 19.45 | 94.70 | 99.70 | 98.96 | 62.97 |
| Sdu_stem_F4 | 85049 | 37.67 | 80685 | 20.11 | 93.72 | 99.60 | 98.61 | 58.93 |
| Sdu_stem_F5 | 83714 | 43.52 | 79319 | 19.95 | 94.85 | 99.72 | 98.98 | 57.15 |
| Sdu_stem_F6 | 81338 | 40.38 | 77253 | 19.10 | 95.06 | 99.63 | 98.63 | 58.19 |
| Sdu_leaf_F1 | 86742 | 43.34 | 82587 | 24.89 | 95.17 | 99.10 | 97.04 | 58.89 |
| Sdu_leaf_F2 | 87127 | 37.89 | 83184 | 21.52 | 94.82 | 99.63 | 98.68 | 55.52 |
| Sdu_leaf_F3 | 86355 | 43.74 | 81956 | 21.12 | 94.78 | 99.44 | 98.01 | 56.01 |
| Sdu_leaf_F4 | 81888 | 42.14 | 77108 | 19.63 | 94.39 | 99.33 | 97.80 | 57.48 |
| Sdu_leaf_F5 | 85841 | 42.92 | 80683 | 24.43 | 93.99 | 97.70 | 92.96 | 59.42 |
| Sdu_leaf_F6 | 84283 | 40.94 | 79555 | 21.03 | 94.16 | 99.20 | 97.37 | 56.88 |
| Sdu_flower_F1 | 81671 | 43.18 | 77789 | 24.37 | 94.91 | 99.17 | 97.33 | 59.84 |
| Sdu_flower_F2 | 87587 | 43.56 | 81140 | 20.21 | 95.47 | 99.32 | 97.83 | 54.44 |
| Sdu_flower_F3 | 80882 | 43.37 | 76622 | 23.94 | 95.21 | 99.13 | 97.16 | 59.79 |
| Sdu_flower_F4 | 87475 | 40.67 | 82905 | 20.44 | 94.98 | 99.72 | 98.93 | 53.89 |
| Sdu_flower_F5 | 75772 | 41.86 | 71848 | 22.55 | 94.75 | 98.47 | 95.07 | 59.94 |
| Sdu_flower_F6 | 86679 | 42.52 | 82495 | 21.22 | 94.87 | 99.63 | 98.77 | 57.48 |
| Sdu_fruit_F1 | 80759 | 40.40 | 76772 | 24.24 | 95.21 | 99.16 | 97.27 | 60.04 |
| Sdu_fruit_F2 | 87050 | 41.83 | 82564 | 25.96 | 90.53 | 99.10 | 97.12 | 59.90 |
| Sdu_fruit_F3 | 75338 | 43.13 | 70609 | 22.21 | 95.50 | 98.26 | 94.53 | 60.03 |
| Sdu_fruit_F4 | 84165 | 43.24 | 79701 | 20.91 | 95.23 | 99.61 | 98.66 | 57.18 |
| Sdu_fruit_F5 | 84769 | 40.18 | 80083 | 20.67 | 90.36 | 99.32 | 97.72 | 45.83 |
| Sdu_fruit_F6 | 80206 | 42.52 | 75157 | 18.99 | 95.34 | 99.51 | 98.29 | 57.87 |

**TABLE S3** Sequencing statistics data of bacterial samples

| Sample | Raw Tags | Raw Bases (M) | Valid Tags | Valid Bases (M) | Valid% | Q20% | Q30% | GC% |
| --- | --- | --- | --- | --- | --- | --- | --- | --- |
| Sdu_root_B1 | 80435 | 41.59 | 74221 | 18.63 | 93.48 | 99.53 | 97.99 | 56.52 |
| Sdu_root_B2 | 84970 | 40.45 | 80142 | 20.12 | 93.76 | 99.60 | 98.22 | 55.82 |
| Sdu_root_B3 | 86646 | 40.79 | 78383 | 19.68 | 92.83 | 98.77 | 95.03 | 55.87 |
| Sdu_root_B4 | 84162 | 43.32 | 79676 | 20.00 | 90.46 | 99.65 | 98.43 | 54.38 |
| Sdu_root_B5 | 83368 | 42.48 | 75657 | 18.99 | 94.32 | 99.61 | 98.29 | 55.26 |
| Sdu_root_B6 | 85775 | 40.22 | 83016 | 20.83 | 92.27 | 99.73 | 98.79 | 54.38 |
| Sdu_stem_B1 | 87379 | 41.27 | 81330 | 20.42 | 94.14 | 99.57 | 98.15 | 54.03 |
| Sdu_stem_B2 | 85125 | 42.70 | 80793 | 20.28 | 94.37 | 99.62 | 98.31 | 54.45 |
| Sdu_stem_B3 | 86794 | 42.41 | 81720 | 20.50 | 94.27 | 99.60 | 98.19 | 53.78 |
| Sdu_stem_B4 | 82768 | 42.59 | 77985 | 19.57 | 93.92 | 99.59 | 98.21 | 55.89 |
| Sdu_stem_B5 | 83629 | 44.00 | 78509 | 19.71 | 93.53 | 99.61 | 98.22 | 53.78 |
| Sdu_stem_B6 | 85590 | 41.24 | 80475 | 20.20 | 93.90 | 99.63 | 98.33 | 54.35 |
| Sdu_leaf_B1 | 87167 | 40.19 | 82227 | 20.64 | 92.38 | 99.61 | 98.28 | 54.16 |
| Sdu_leaf_B2 | 82980 | 42.92 | 78462 | 19.70 | 93.75 | 99.64 | 98.36 | 53.94 |
| Sdu_leaf_B3 | 86153 | 43.58 | 81229 | 20.39 | 92.67 | 99.62 | 98.32 | 54.22 |
| Sdu_leaf_B4 | 81849 | 40.14 | 76983 | 19.32 | 93.83 | 99.62 | 98.32 | 53.97 |
| Sdu_leaf_B5 | 87437 | 43.72 | 82322 | 20.66 | 94.15 | 99.61 | 98.27 | 54.86 |
| Sdu_leaf_B6 | 80272 | 40.92 | 75317 | 18.90 | 94.05 | 99.59 | 98.22 | 53.58 |
| Sdu_flower_B1 | 81586 | 43.08 | 75740 | 19.01 | 94.28 | 99.44 | 97.60 | 56.73 |
| Sdu_flower_B2 | 80909 | 41.49 | 75862 | 19.04 | 94.56 | 99.57 | 98.10 | 55.84 |
| Sdu_flower_B3 | 83181 | 43.58 | 77758 | 19.52 | 94.33 | 99.45 | 97.60 | 54.63 |
| Sdu_flower_B4 | 87159 | 42.80 | 80773 | 20.28 | 94.02 | 99.58 | 98.16 | 55.48 |
| Sdu_flower_B5 | 85839 | 41.81 | 80474 | 20.20 | 93.88 | 99.58 | 98.10 | 52.56 |
| Sdu_flower_B6 | 80377 | 41.38 | 74252 | 18.65 | 94.22 | 99.58 | 98.13 | 55.08 |
| Sdu_fruit_B1 | 82479 | 43.40 | 77448 | 19.44 | 94.15 | 99.53 | 97.89 | 54.37 |
| Sdu_fruit_B2 | 87991 | 42.56 | 82296 | 20.65 | 94.91 | 99.58 | 98.05 | 53.85 |
| Sdu_fruit_B3 | 85178 | 43.69 | 80002 | 20.08 | 93.08 | 99.60 | 98.19 | 54.22 |
| Sdu_fruit_B4 | 84817 | 42.89 | 79955 | 20.07 | 96.78 | 99.65 | 98.36 | 54.41 |
| Sdu_fruit_B5 | 85401 | 41.68 | 80591 | 20.23 | 90.75 | 99.65 | 98.41 | 53.76 |
| Sdu_fruit_B6 | 82544 | 42.08 | 77705 | 19.50 | 94.67 | 99.58 | 98.07 | 53.85 |

**TABLE S4** The top 30 significant correlations for each comparison group

| Comparison group | Genus | Metabolites | rho | p_value |
| --- | --- | --- | --- | --- |
| Fruits and roots | *Hyphomicrobiaceae_unclassified* | D-Alloisoleucine | -0.97 | 0.00 |
| (bacteria) | *Acidothermus* | Hydroxyphenylacetylglycine | -0.94 | 0.00 |
|  | *Rhodoplanes* | Gibberellin A53 | -0.94 | 0.00 |
|  | *Acidothermus* | Leu-Phe | -0.94 | 0.00 |
|  | *Hyphomicrobiaceae_unclassified* | Epigallocatechin-3-gallate | -0.93 | 0.00 |
|  | *Rhizobiales_unclassified* | Isonicotinic acid | -0.93 | 0.00 |
|  | *Rhizobiales_unclassified* | 6-Oxopiperidine-2-carboxylic acid | -0.93 | 0.00 |
|  | *Rhizobiales_unclassified* | Val-Val | -0.92 | 0.00 |
|  | *Rhizobiales_unclassified* | 2,3-Dihydroxyvaleric acid | -0.92 | 0.00 |
|  | *Rhizobiales_unclassified* | Glyceric acid | -0.92 | 0.00 |
|  | *Rhizobiales_unclassified* | Ile-Ala | -0.92 | 0.00 |
|  | *Rhodoplanes* | 3-Methylglutaconic acid | -0.92 | 0.00 |
|  | *Rhodoplanes* | 3,4-Methylenesebacic acid | -0.92 | 0.00 |
|  | *Rhodoplanes* | 3-Indoleacetic acid | -0.92 | 0.00 |
|  | *Acidothermus* | Echinocystic acid | -0.92 | 0.00 |
|  | *Acidothermus* | 3-Hydroxy-3-methylglutaric acid | -0.92 | 0.00 |
|  | *Hyphomicrobiaceae_unclassified* | Naphthoherniarin | -0.92 | 0.00 |
|  | *Hyphomicrobiaceae_unclassified* | N4-Acetylsulfadiazine | -0.92 | 0.00 |
|  | *Chloroplast_unclassified* | Dihydroxyacetone | -0.91 | 0.00 |
|  | *Chloroplast_unclassified* | Mefenorex | -0.91 | 0.00 |
|  | *Rhizobiales_unclassified* | Trinexapac | -0.91 | 0.00 |
|  | *Rhizobiales_unclassified* | 2-Methyl-3-ketovaleric acid | -0.91 | 0.00 |
|  | *Hyphomicrobiaceae_unclassified* | Dihydroxyacetone | 0.94 | 0.00 |
|  | *Rhodoplanes* | Ethyl vanillin | 0.93 | 0.00 |
|  | *Acidothermus* | DGDG 36:5 | 0.92 | 0.00 |
|  | *Chloroplast_unclassified* | 2-Linoleoylglycerol | 0.91 | 0.00 |
|  | *Acidothermus* | (7'R)-(+)-Lyoniresinol 9'-glucoside | 0.91 | 0.00 |
|  | *Chloroplast_unclassified* | LysoPI 18:2 | 0.91 | 0.00 |
|  | *Chloroplast_unclassified* | beta-D-Glucopyranosyl-11-hydroxyjasmonic acid | 0.91 | 0.00 |
|  | *Rhizobiales_unclassified* | Vanylglycol | 0.91 | 0.00 |
| Fruits and roots | *Branch06_unclassified* | Quercetin-3-O-glucuronide | 0.81 | 0.00 |
| (fungi) | *Candida* | (R)-2,3-Dihydroxy-isovalerate | 0.80 | 0.00 |
| Fruits and stems | *Sphingomonas* | Arbutin | -0.92 | 0.00 |
| (bacteria) | *Sphingomonas* | Methionine | -0.92 | 0.00 |
|  | *Ralstonia* | Dibenzyl disulfide | -0.91 | 0.00 |
|  | *Sphingomonas* | (-)-Citramalic acid | -0.91 | 0.00 |
|  | *Sphingomonas* | Leu-Gly | -0.91 | 0.00 |
|  | *Sphingomonas* | Cyclic adenosine diphosphate ribose | -0.91 | 0.00 |
|  | *Ralstonia* | Ala-Val | 0.95 | 0.00 |
|  | *Ralstonia* | Leu-Glu | 0.94 | 0.00 |
|  | *Ralstonia* | D-(+)-Pantothenic acid | 0.94 | 0.00 |
|  | *Bradyrhizobiaceae_unclassified* | (2E,6E)-1-Hydroxy-2,6,10-farnesatrien-9-one | 0.94 | 0.00 |
|  | *Ralstonia* | Lysyl-Leucine | 0.93 | 0.00 |
|  | *Ralstonia* | Phenylalanine | 0.93 | 0.00 |
|  | *Ralstonia* | Leucyl-Isoleucine | 0.93 | 0.00 |
|  | *Ralstonia* | Uridine | 0.92 | 0.00 |
|  | *Ralstonia* | Ile-Ala | 0.92 | 0.00 |
|  | *Ralstonia* | Ile-Ser | 0.92 | 0.00 |
|  | *Ralstonia* | Leu-Pro | 0.92 | 0.00 |
|  | *Bradyrhizobiaceae_unclassified* | Norecasantalic acid | 0.92 | 0.00 |
|  | *Bradyrhizobiaceae_unclassified* | 6-Methoxy-7-hydroxycoumarin | 0.92 | 0.00 |
|  | *Ralstonia* | 3-Hydroxy-3-methylglutaric acid | 0.92 | 0.00 |
|  | *Ralstonia* | Val-Val | 0.92 | 0.00 |
|  | *Ralstonia* | Aspartyl-Isoleucine | 0.91 | 0.00 |
|  | *Ralstonia* | N-[4'-hydroxy-(E)-cinnamoyl]-L-aspartic acid | 0.91 | 0.00 |
|  | *Ralstonia* | Leu-Gly | 0.91 | 0.00 |
|  | *Ralstonia* | Adenine | 0.91 | 0.00 |
|  | *Ralstonia* | Ser-Leu | 0.91 | 0.00 |
|  | *Ralstonia* | 4-Acetamidobutanoic acid | 0.91 | 0.00 |
|  | *Ralstonia* | Threonine | 0.91 | 0.00 |
|  | *Sphingomonas* | Sinapoyl aldehyde | 0.91 | 0.00 |
|  | *Sphingomonas* | (R)-Bitalin A | 0.91 | 0.00 |
| Fruits and stems | *Fungi_unclassified* | Sinapoyl aldehyde | -0.95 | 0.00 |
| (fungi) | *Fungi_unclassified* | (+)-cis-5,6-Dihydro-5-hydroxy-4-methoxy-6-(2-phenylethyl)-2H-pyran-2-one | -0.94 | 0.00 |
|  | *Diaporthe* | N-Acetyl-L-glutamate 5-semialdehyde | -0.94 | 0.00 |
|  | *Fungi_unclassified* | (R)-Bitalin A | -0.94 | 0.00 |
|  | *Fungi_unclassified* | 2-Anisaldehyde | -0.94 | 0.00 |
|  | *Asterotremella* | PI 8:0 | -0.94 | 0.00 |
|  | *Amphisphaeriaceae_unclassified* | Asparaginyl-Isoleucine | -0.93 | 0.00 |
|  | *Amphisphaeriaceae_unclassified* | 3',5'-Cyclic AMP | -0.93 | 0.00 |
|  | *Amphisphaeriaceae_unclassified* | Gln-Ile | -0.93 | 0.00 |
|  | *Amphisphaeriaceae_unclassified* | 1,4-Diaminonaphthalene | -0.93 | 0.00 |
|  | *Fungi_unclassified* | Pyrenochaetic acid C_120095 | -0.92 | 0.00 |
|  | *Ascomycota_unclassified* | Nandrolone | 0.98 | 0.00 |
|  | *Fungi_unclassified* | Thiopropionic acid, S-ethyl ester | 0.97 | 0.00 |
|  | *Fungi_unclassified* | Adenine | 0.96 | 0.00 |
|  | *Fungi_unclassified* | (-)-Citramalic acid | 0.95 | 0.00 |
|  | *Fungi_unclassified* | 3-Hydroxy-3-methylglutaric acid | 0.95 | 0.00 |
|  | *Fungi_unclassified* | Leu-Gly | 0.95 | 0.00 |
|  | *Fungi_unclassified* | Adenosine | 0.95 | 0.00 |
|  | Fungi_unclassified | Leu-Thr | 0.94 | 0.00 |
|  | *Sordariomycetes_unclassified* | xi-8-Hydroxyhexadecanedioic acid | 0.94 | 0.00 |
|  | *Fungi_unclassified* | Leu-Glu | 0.94 | 0.00 |
|  | *Fungi_unclassified* | Ile-Ala | 0.94 | 0.00 |
|  | *Fungi_unclassified* | Ile-Ser | 0.94 | 0.00 |
|  | *Sordariomycetes_unclassified* | Biflorin | 0.94 | 0.00 |
|  | *Sordariomycetes_unclassified* | Quinone | 0.94 | 0.00 |
|  | *Fungi_unclassified* | Leu-Ala | 0.93 | 0.00 |
|  | *Fungi_unclassified* | Phe-Pro | 0.93 | 0.00 |
|  | *Fungi_unclassified* | Arg-Leu | 0.93 | 0.00 |
|  | *Dothideomycetes_unclassified* | Mollugin | 0.93 | 0.00 |
|  | *Fungi_unclassified* | Lysyl-Leucine | 0.92 | 0.00 |
| Fruits and leaves | *Chloroplast_unclassified* | LysoPC 18:3 | 0.93 | 0.00 |
| (bacteria) | *Chloroplast_unclassified* | 3-(Carboxymethyl)-3-hydroxypentanedioic acid | 0.90 | 0.00 |
|  | *Chloroplast_unclassified* | 8(9)-Epoxy-5Z,11Z,14Z-eicosatrienoic acid, methyl ester | 0.87 | 0.00 |
|  | *Bradyrhizobiaceae_unclassified* | Adenine | 0.86 | 0.00 |
|  | *Chloroplast_unclassified* | p-Ethylacetophenone | 0.85 | 0.00 |
|  | *Chloroplast_unclassified* | Isothankunic acid | 0.85 | 0.00 |
|  | *Chloroplast_unclassified* | Octadecanedioic acid | 0.84 | 0.00 |
|  | *Bradyrhizobiaceae_unclassified* | 3-[4-Hydroxy-3-(3-methyl-2-butenyl)phenyl]-2-propenal | 0.84 | 0.00 |
|  | *Chloroplast_unclassified* | Pyridoxine | 0.83 | 0.00 |
|  | *Corynebacterium* | Phenylmethyl benzeneacetate | 0.83 | 0.00 |
|  | *Corynebacterium* | Methuyl tanshinonate | 0.83 | 0.00 |
|  | *Chloroplast_unclassified* | Furanone A | 0.83 | 0.00 |
|  | *Chloroplast_unclassified* | 2,2,6,6-Tetramethyl-4-piperidinone | 0.83 | 0.00 |
|  | *Corynebacterium* | 4-Hydroxyphenyl-2-propionic acid | 0.82 | 0.00 |
|  | *Corynebacterium* | trans-3,5-Dimethoxy-4-hydroxycinnamaldehyde | 0.82 | 0.00 |
|  | *Corynebacterium* | 3-O-p-Coumaroylquinic acid | 0.82 | 0.00 |
|  | *Corynebacterium* | 3'-(6''-Galloylglucosyl)-phloroacetophenone | 0.82 | 0.00 |
|  | *Corynebacterium* | Coumarin | 0.82 | 0.00 |
|  | *Bradyrhizobiaceae_unclassified* | Phenylacetic acid | 0.81 | 0.00 |
|  | *Chloroplast_unclassified* | Phosphoric acid | -0.90 | 0.00 |
|  | *Chloroplast_unclassified* | Gibberellin A110 | -0.87 | 0.00 |
|  | *Chloroplast_unclassified* | Leu-Arg | -0.86 | 0.00 |
|  | *Bradyrhizobiaceae_unclassified* | LysoPE 20:1 | -0.86 | 0.00 |
|  | *Chloroplast_unclassified* | Tramadol | -0.85 | 0.00 |
|  | *Bradyrhizobiaceae_unclassified* | Leu-Ser | -0.83 | 0.00 |
|  | *Chloroplast_unclassified* | Xanthosine | -0.83 | 0.00 |
|  | *Corynebacterium* | 12-Oxo-20-trihydroxy-leukotriene B4 | -0.83 | 0.00 |
|  | Chloroplast_unclassified | O-Desmethyl-cis-tramadol | -0.83 | 0.00 |
|  | *Chloroplast_unclassified* | Ile-Ser | -0.82 | 0.00 |
|  | *Chloroplast_unclassified* | Leu-Pro | -0.81 | 0.00 |
| Fruits and leaves | *Sordariomycetes_unclassified* | Acetone cyanohydrin | -0.93 | 0.00 |
| (fungi) | *Guignardia* | 5-Amino-1-pentanol | -0.92 | 0.00 |
|  | *Glomerella* | D-Asparagine | -0.92 | 0.00 |
|  | *Guignardia* | D-Asparagine | -0.92 | 0.00 |
|  | *Guignardia* | LysoPC 16:0 | -0.92 | 0.00 |
|  | *Guignardia* | DL-Pipecolinic acid | -0.91 | 0.00 |
|  | *Sordariomycetes_unclassified* | Pentanenitrile | -0.91 | 0.00 |
|  | *Ophioceras* | 12-Oxo-20-trihydroxy-leukotriene B4 | -0.90 | 0.00 |
|  | *Guignardia* | Val-Asp | -0.90 | 0.00 |
|  | *Paraconiothyrium* | Propyl 2,4-decadienoate | -0.89 | 0.00 |
|  | *Paraconiothyrium* | 18-BETA-GLYCYRRHETINIC ACID471-53-4 | -0.89 | 0.00 |
|  | *Guignardia* | PI 7:0 | 0.98 | 0.00 |
|  | *Guignardia* | (2E,6E)-1-Hydroxy-2,6,10-farnesatrien-9-one | 0.97 | 0.00 |
|  | *Guignardia* | Adenine | 0.94 | 0.00 |
|  | *Guignardia* | Zerumbone | 0.94 | 0.00 |
|  | *Guignardia* | 1-(2,4-Dihydroxyphenyl)-1-butanone | 0.92 | 0.00 |
|  | *Guignardia* | Cubebin | 0.92 | 0.00 |
|  | *Guignardia* | 5Z,8Z,14Z-Eicosatrienoic acid | 0.91 | 0.00 |
|  | *Guignardia* | D-Ribulose | 0.90 | 0.00 |
|  | *Guignardia* | (E)-3-(2-Methylpropylidene)-1(3H)-isobenzofuranone | 0.90 | 0.00 |
|  | *Glomerella* | Teucrin A | 0.90 | 0.00 |
|  | *Paraconiothyrium* | 2'-Deoxyadenosine | 0.90 | 0.00 |
|  | *Paraconiothyrium* | Crotonic acid | 0.90 | 0.00 |
|  | *Paraconiothyrium* | L-Asparagine | 0.90 | 0.00 |
|  | *Ophioceras* | Phenylmethyl benzeneacetate | 0.90 | 0.00 |
|  | *Guignardia* | LysoDGTS 18:2 | 0.90 | 0.00 |
|  | *Guignardia* | 3-(4-Methoxyphenyl)-2-methyl-2-propenal | 0.90 | 0.00 |
|  | *Paraconiothyrium* | rac-Glycerol 3-phosphoate | 0.89 | 0.00 |
|  | *Ophioceras* | 3-O-p-Coumaroylquinic acid | 0.89 | 0.00 |
|  | *Ophioceras* | 4-Hydroxyphenyl-2-propionic acid | 0.89 | 0.00 |
| Fruits and flowers | *Enterobacter* | Uridine | -0.98 | 0.00 |
| (bacteria) | *Ralstonia* | Gallic acid 3-O-(6-galloylglucoside) | -0.94 | 0.00 |
|  | *Enterobacter* | N-[4'-hydroxy-(E)-cinnamoyl]-L-aspartic acid | -0.94 | 0.00 |
|  | *Ralstonia* | 6-Methoxy-7-hydroxycoumarin | -0.94 | 0.00 |
|  | *Ralstonia* | 7,8,2'-Trihydroxyflavone | -0.94 | 0.00 |
|  | *Rhodococcus* | (-)-epicatechin-3'-O-glucuronide | -0.94 | 0.00 |
|  | *Ralstonia* | 4-Hydroxy-2,6-dimethylaniline | -0.94 | 0.00 |
|  | *Enterobacter* | L-Glutamine | -0.94 | 0.00 |
|  | *Enterobacter* | L-5-Oxoproline | -0.94 | 0.00 |
|  | *Actinomycetospora* | Arg-Leu | -0.94 | 0.00 |
|  | *Sphingomonas* | Arg-Leu | -0.94 | 0.00 |
|  | *Ralstonia* | Methyl 6-O-galloyl-beta-D-glucopyranoside | -0.93 | 0.00 |
|  | *Ralstonia* | (+)-Usnic acid | -0.93 | 0.00 |
|  | *P3OB-42* | N-Desmethylmirtazapine | -0.93 | 0.00 |
|  | *Jatrophihabitans* | dimethadione | -0.93 | 0.00 |
|  | *Labrys* | (+)-cis-5,6-Dihydro-5-hydroxy-4-methoxy-6-(2-phenylethyl)-2H-pyran-2-one | -0.93 | 0.00 |
|  | *Actinomycetospora* | 12,13-Dihydroxy-9Z-octadecenoic acid | 0.95 | 0.00 |
|  | *Sphingomonas* | 12,13-Dihydroxy-9Z-octadecenoic acid | 0.94 | 0.00 |
|  | *Sphingomonas* | Eriodictyol | 0.94 | 0.00 |
|  | *Methylobacterium* | Eriodictyol | 0.94 | 0.00 |
|  | *P3OB-42* | LysoPC 17:0 | 0.94 | 0.00 |
|  | *Actinomycetospora* | Chalconaringenin | 0.94 | 0.00 |
|  | *Sphingomonas* | Chalconaringenin | 0.94 | 0.00 |
|  | *Ralstonia* | Histidinyl-Isoleucine | 0.93 | 0.00 |
|  | *Enterobacter* | Dihydrokaempferol | 0.93 | 0.00 |
|  | *P3OB-42* | Echinocystic acid | 0.93 | 0.00 |
|  | *Jatrophihabitans* | 2,4-Di-tert-butylphenol | 0.93 | 0.00 |
|  | *Jatrophihabitans* | DL-2-Aminooctanoic acid | 0.93 | 0.00 |
|  | *P3OB-42* | Phe-His | 0.93 | 0.00 |
|  | *Jatrophihabitans* | Pipemidic acid | 0.93 | 0.00 |
| Fruits and flowers  (fungi) | *Xylaria* | Nandrolone | -0.83 | 0.00 |

**TABLE S5** GenBank accession number of 41 fungi and 28 bacterial

|  | Endophytes | Genus | Accession number |
| --- | --- | --- | --- |
| 1 | S23 | *Cladosporium* sp. | OR018836 |
| 2 | S18 | *Cladosporium halotolerans* | OR018887 |
| 3 | FR16 | *Cladosporium* sp. | OR018888 |
| 4 | S7 | *Xylaria plebeja* | OR018890 |
| 5 | S9 | *Xylaria* sp. | OR018891 |
| 6 | S10 | *Xylaria* sp. | OR019764 |
| 7 | S12 | *Xylaria* sp. | OR019761 |
| 8 | L3 | *Xylaria grammica* | OR019769 |
| 9 | FL23 | *Xylaria papulis* | OR019001 |
| 10 | R16 | *Xylaria schweinitzii* | OR018998 |
| 11 | S1 | *Arthopyrenia* sp. | OR019630 |
| 12 | S11 | *Nemania bipapillata* | OR019652 |
| 13 | S14 | *Hypoxylon* sp. | OR019672 |
| 14 | S17 | *Stachybotrys chlorohalonata* | OR019671 |
| 15 | FL32 | *Nigrospora* sp. | OR019682 |
| 16 | FR23 | *Nigrospora* sp. | OR019687 |
| 17 | FR31 | *Nigrospora oryzae* | OR019686 |
| 18 | FR32 | *Nigrospora* sp. | OR019695 |
| 19 | L48 | *Nigrospora* sp. | OR019705 |
| 20 | L39 | *Daldinia eschscholtzii* | OR019748 |
| 21 | FR9 | *Daldinia* sp. | OR019777 |
| 22 | FR27 | *Diaporthe* sp. | OR019802 |
| 23 | L36 | *Diaporthe phaseolorum* | OR019812 |
| 24 | S8 | *Penicillium paxilli* | OR019811 |
| 25 | FR3 | *Penicillium herquei* | OR019835 |
| 26 | L17 | *Penicillium chrysogenum* | OR019833 |
| 27 | L21 | *Penicillium copticola* | OR019832 |
| 28 | L7 | *Phomopsis* sp. | OR019850 |
| 29 | FL16 | *Phomopsis* sp. | OR019836 |
| 30 | FR8 | *Phomopsis* sp. | OR019880 |
| 31 | L8 | *Colletotrichum siamense* | OR019881 |
| 32 | L9 | *Colletotrichum gloeosporioides* | OR019878 |
| 33 | L22 | *Colletotrichum fructicola* | OR020016 |
| 34 | FL1 | *Colletotrichum* sp. | OR020174 |
| 35 | R1 | *Aspergillus* sp. | OR020175 |
| 36 | R4 | *Aspergillus* sp. | OR020179 |
| 37 | FL18 | *Zopfiella* sp. | OR020177 |
| 38 | FL11 | *Byssochlamys spectabilis* | OR020594 |
| 39 | FL27 | *Neofusicoccum parvum* | OR020595 |
| 40 | S3 | *Lambertella* sp. | OR020593 |
| 41 | L55 | *Mucor* sp. | OR020602 |
| 42 | fr5 | *Protaetiibacter* sp. | OR030132 |
| 43 | fl7 | *Protaetiibacter* sp. | OR030320 |
| 44 | fl27 | *Protaetiibacter* sp. | OR030331 |
| 45 | fl12 | *Bacillus* sp. | OR039450 |
| 46 | fl21 | *Bacillus* sp. | OR039554 |
| 47 | s3 | *Bacillus* sp. | OR030135 |
| 48 | s4 | *Microbacterium* sp. | OR039453 |
| 49 | fl9 | *Microbacterium* sp. | OR039533 |
| 50 | fl24 | *Streptomyces* sp. | OR030134 |
| 51 | s1 | *Streptomyces* sp. | OR039454 |
| 52 | r5 | *Streptomyces* sp. | OR039451 |
| 53 | fl8 | *Streptomyces* sp. | OR039456 |
| 54 | s9 | *Streptomyces* sp. | OR039464 |
| 55 | fl16 | *Streptomyces* sp. | OR039535 |
| 56 | fl17 | *Streptomyces* sp. | OR039539 |
| 57 | fr7 | *Streptomyces* sp. | OR039541 |
| 58 | r2 | *Mycolicibacterium* sp. | OR030160 |
| 59 | s8 | *Gordonia* sp. | OR039536 |
| 60 | s7 | *Gordonia* sp. | OR039538 |
| 61 | fr10 | *Brevundimonas* sp. | OR039452 |
| 62 | fr1 | *Pseudonocardia* sp. | OR030348 |
| 63 | s5 | *Rhodococcus* sp. | OR030335 |
| 64 | fr6 | *Sphingomonas* sp. | OR039455 |
| 65 | l1 | *Microlunatus* sp. | OR039462 |
| 66 | r7 | *Kitasatospora* sp. | OR039457 |
| 67 | l6 | *Paracoccus* sp. | OR039461 |
| 68 | fr8 | *Agrococcus* sp. | OR039542 |
| 69 | fr9 | *Methylobacterium* sp. | OR039540 |


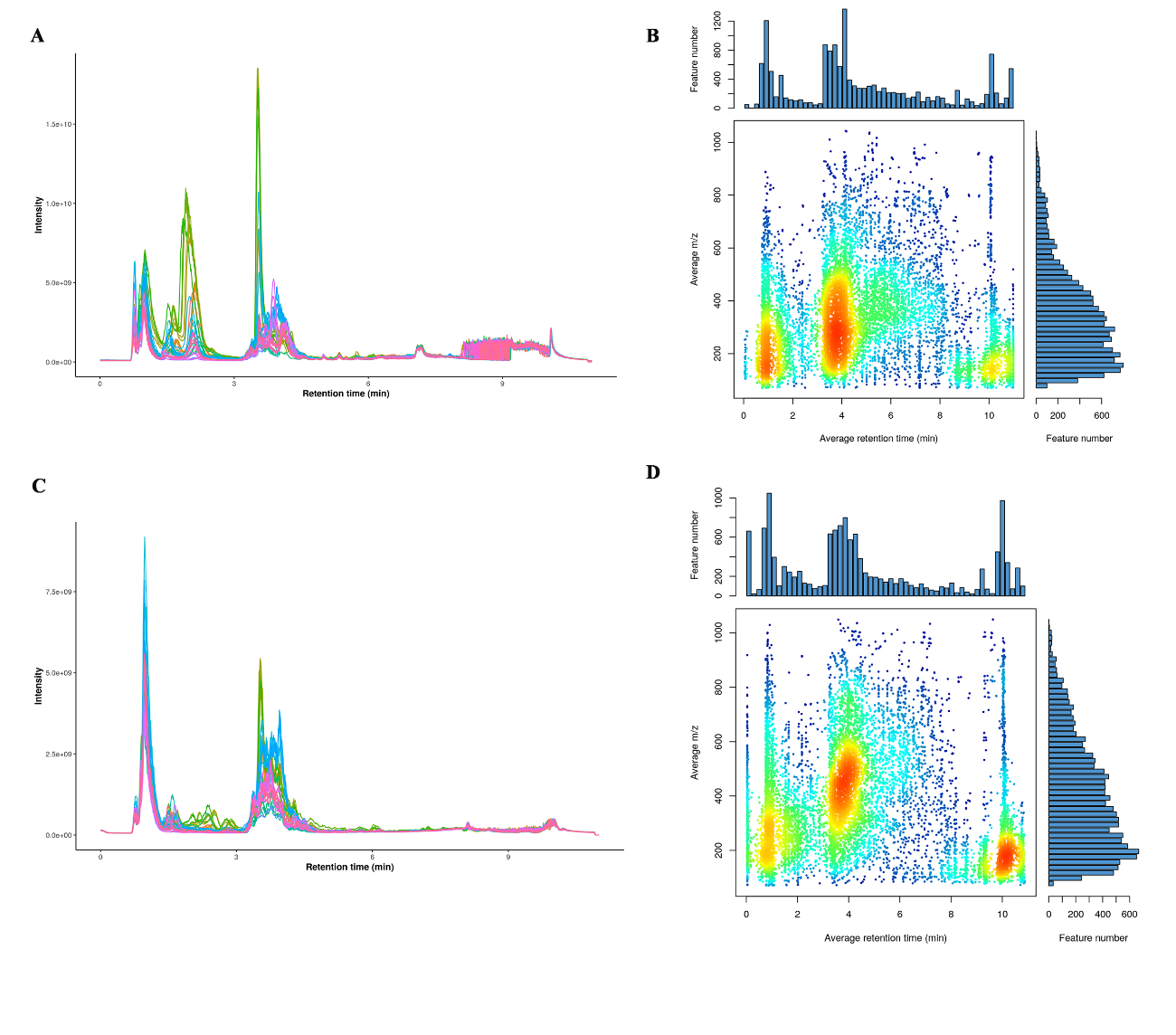


**FIGURE S1** Total ion chromatogram and metabolite m/z-rt distribution map (A-B, positive ionization mode; C-D, negative ionization mode)


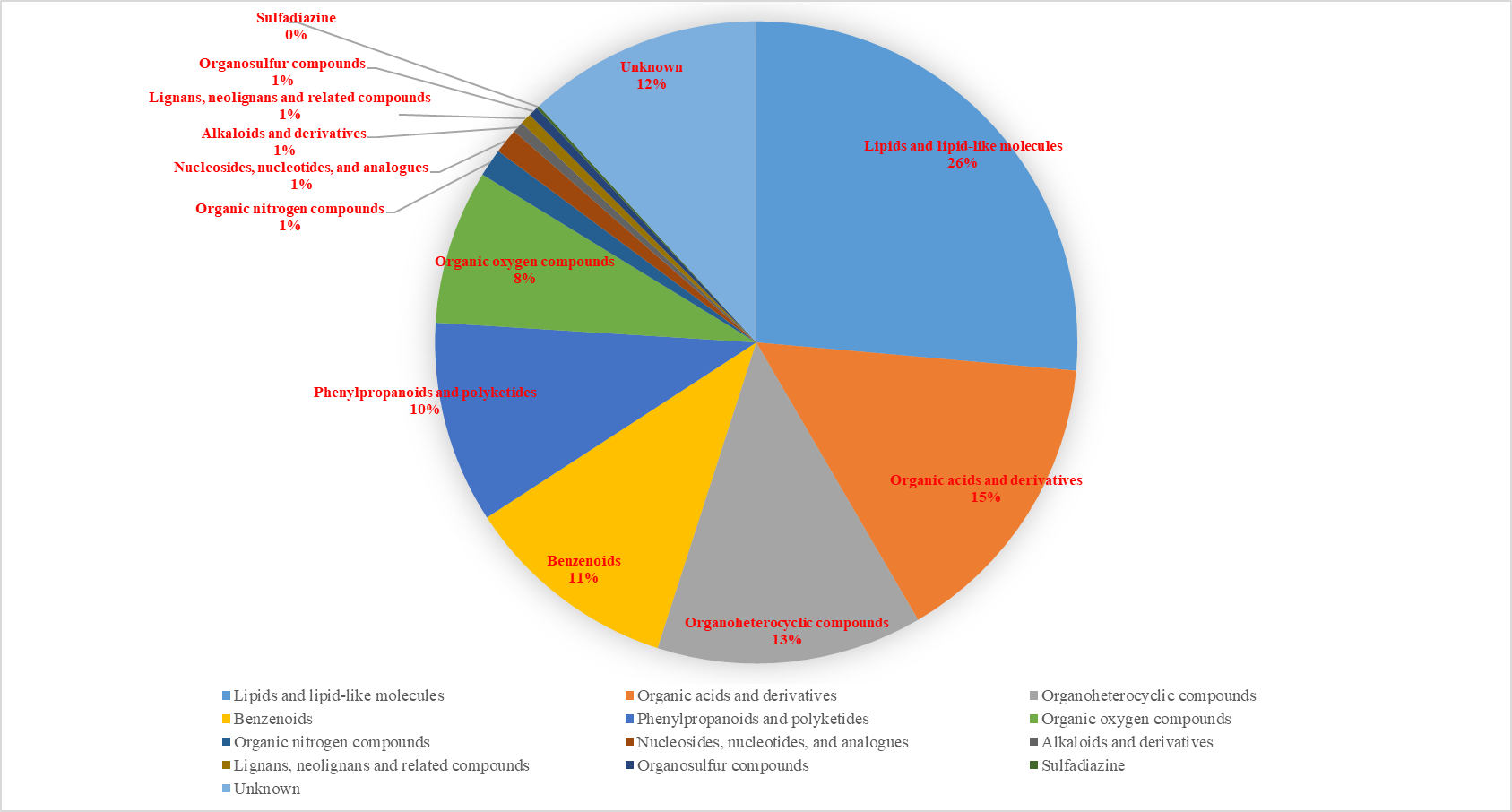


**FIGURE S2** Pie chart of metabolites classification in 5 tissues of *Synsepalum dulcificum*


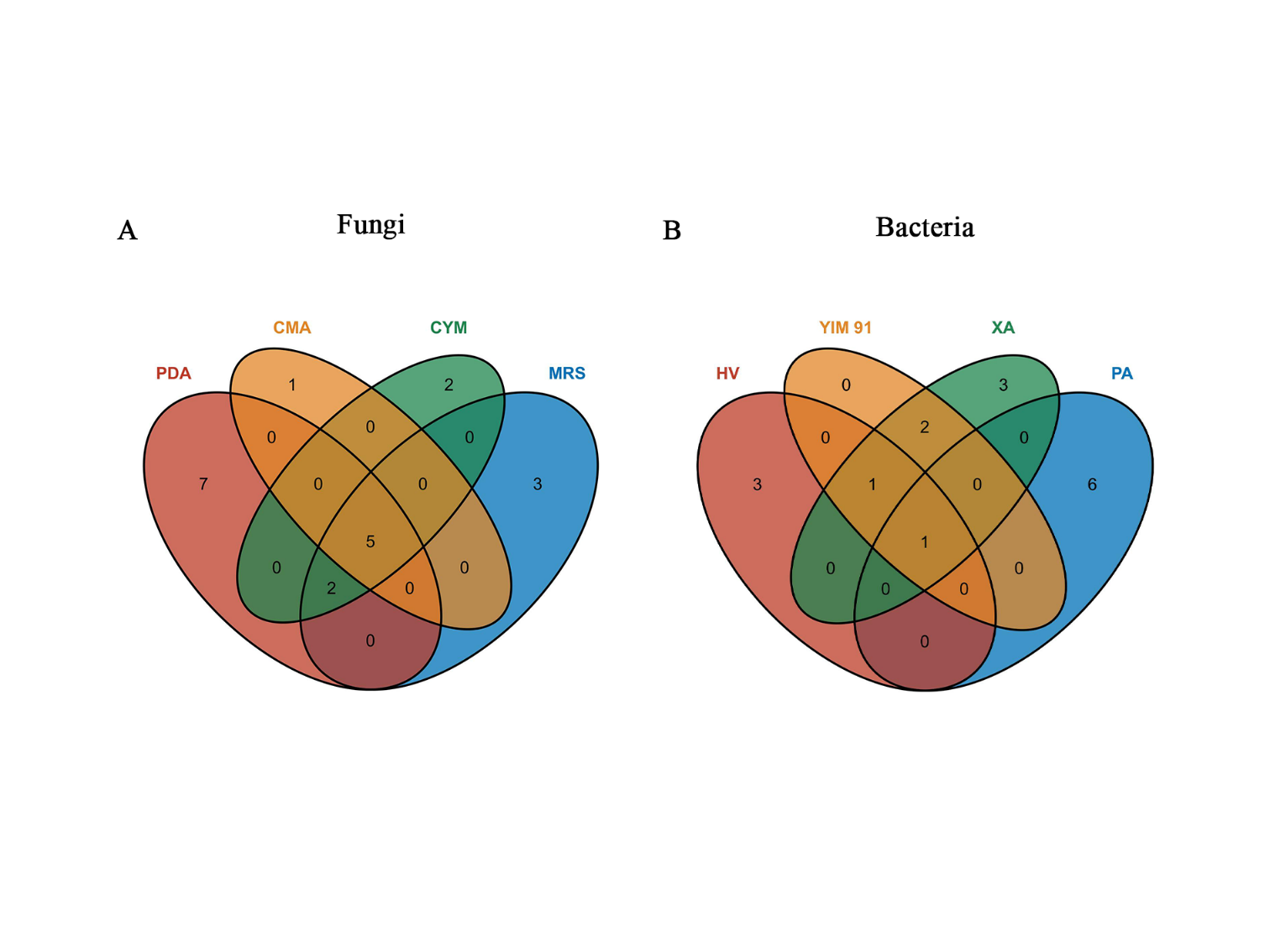


**FIGURE S3** Venn diagram of endophytes isolated from different media (**A**: fungi, **B**: bacteria).


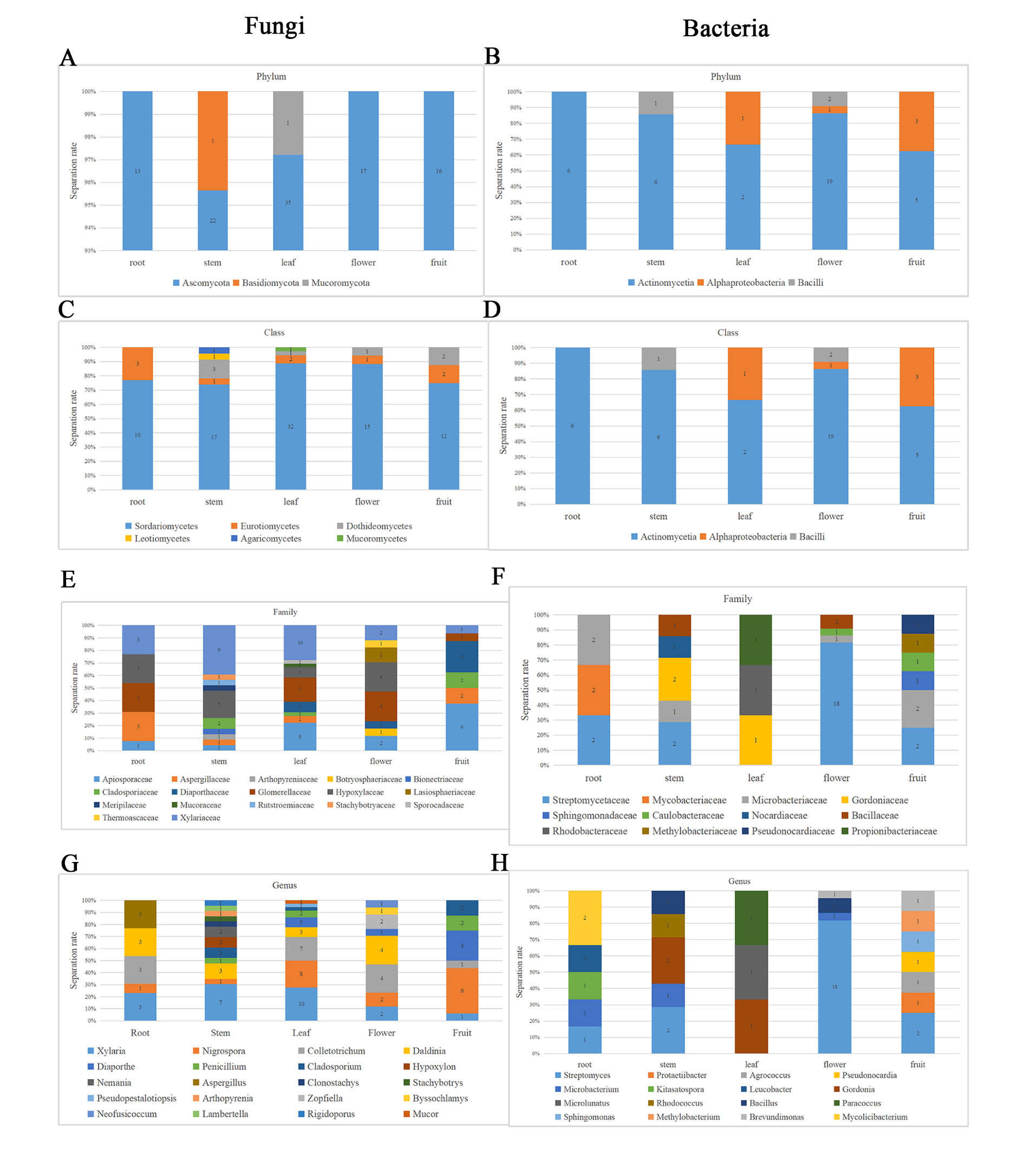


**FIGURE S4** Mean relative abundance of the abundant phylum, class, family, and genus in culturable fungi **(A, C, E, G)** and bacteria **(B, D, F, H)**.


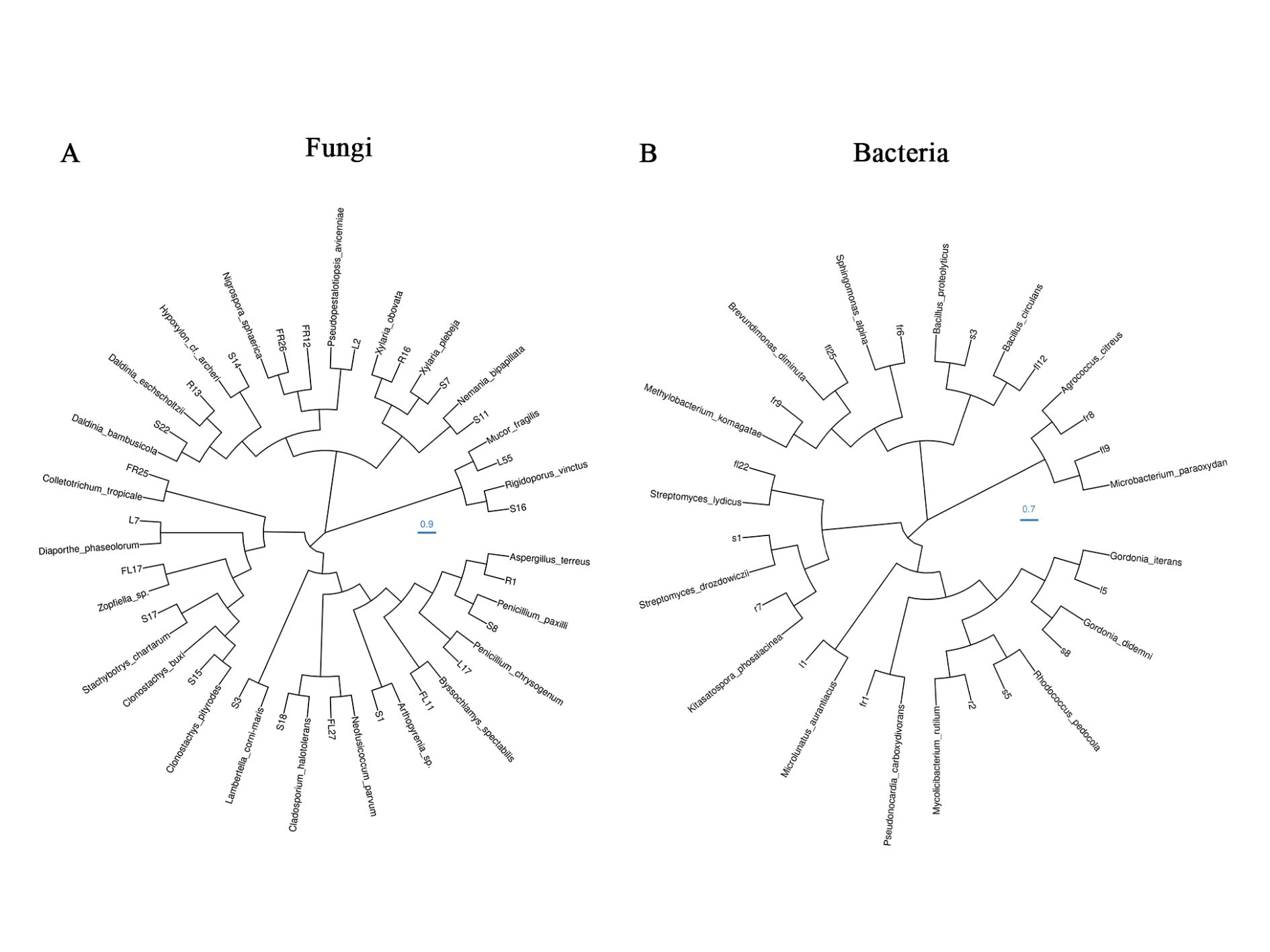


**FIGURE S5** Phylogenetic tree of partial sequence of endophytes from *S*. *dulcificum* (**A**: fungi; **B**: bacteria).


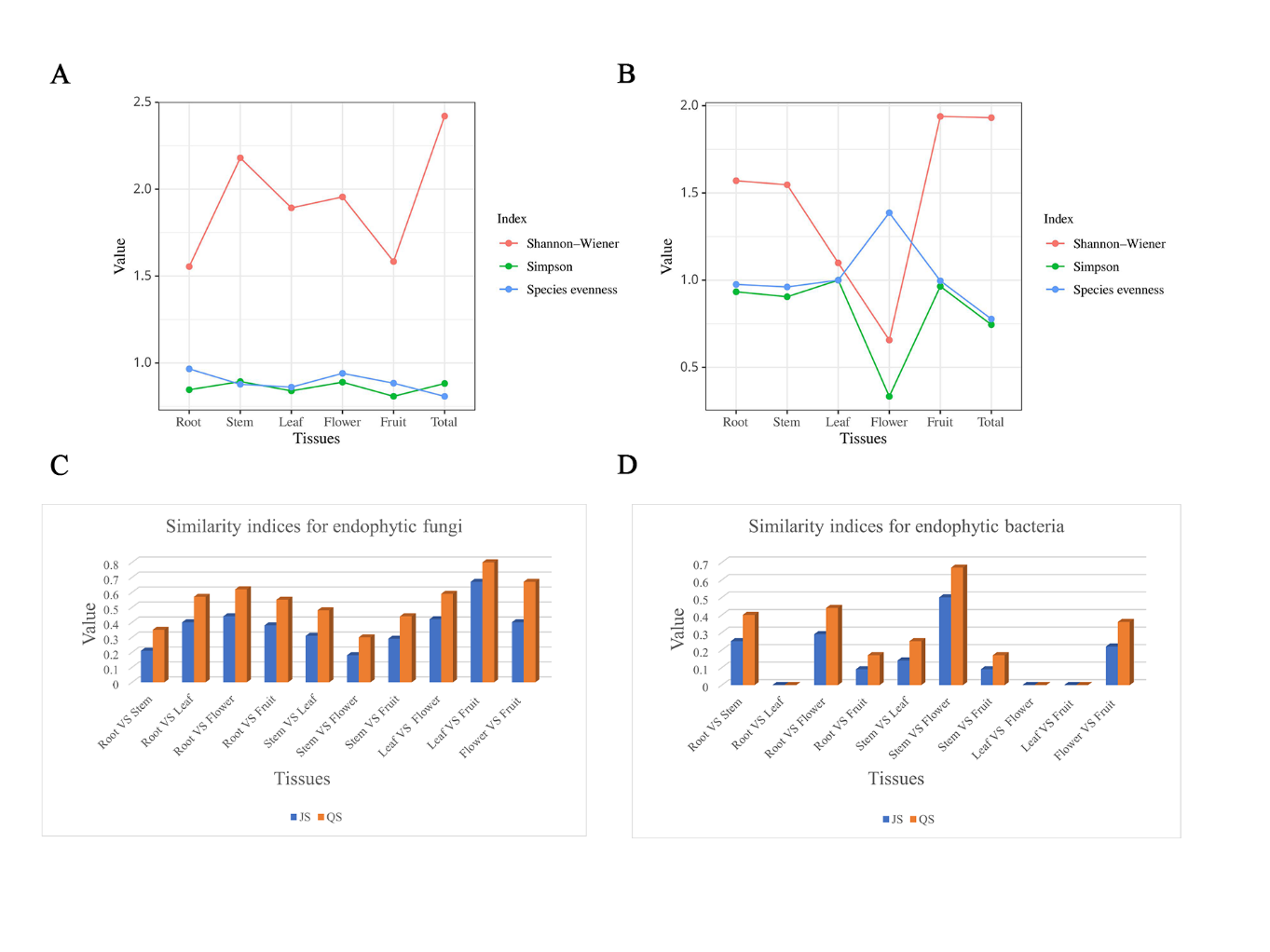


**FIGURE S6** Alpha diversity, Sorensen’s and Jaccard’s index of similarity of culturable fungi **(A, C)** and bacteria **(B, D)**.


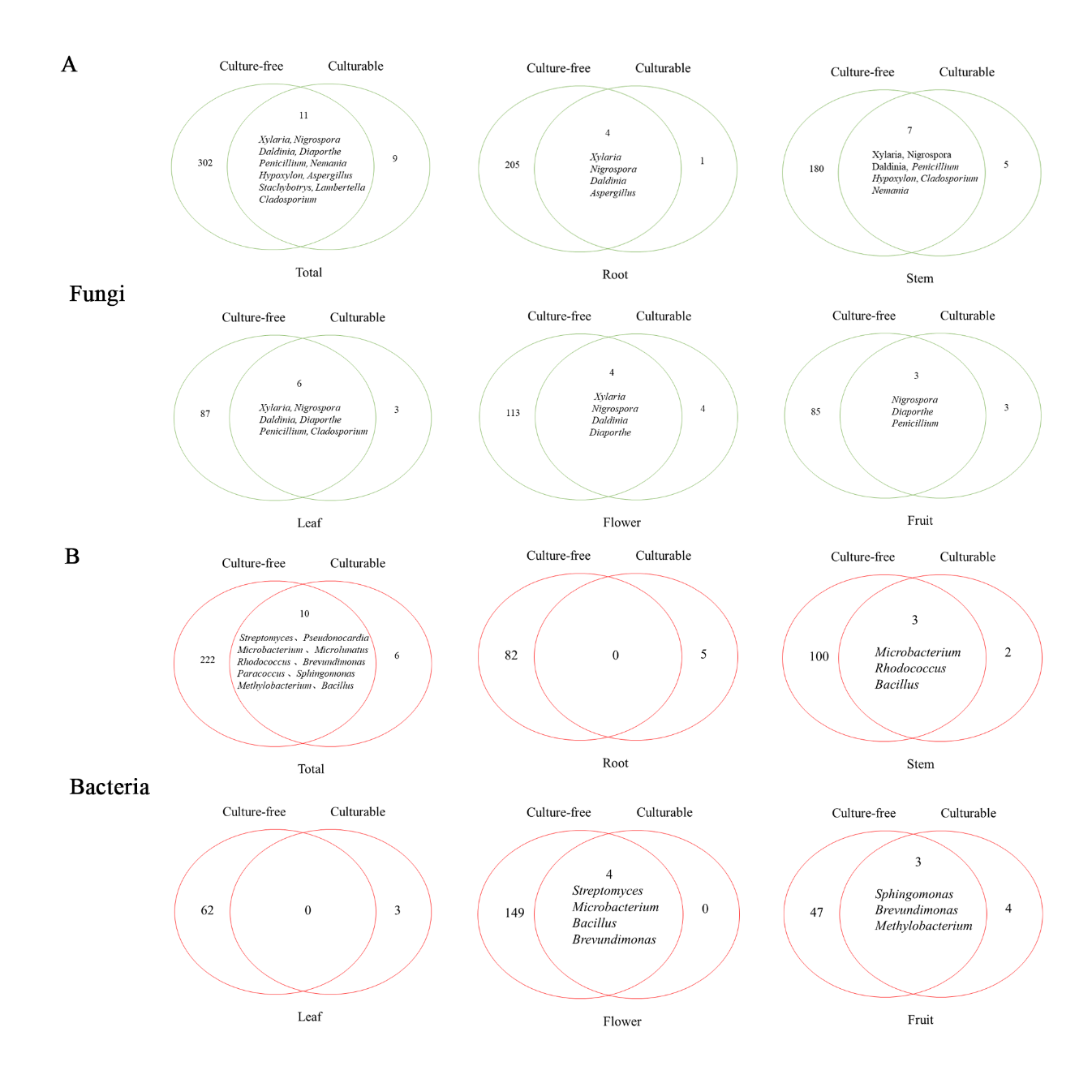


**FIGURE** **S7** Comparison of culture-free uncultured and culturable strains in different tissues (**A**: fungi; **B**: bacteria).
